# Identification of Hub Neutrophil Coexpressed Genes in Acute Pancreatitis *via* Machine Learning

**DOI:** 10.2174/0118715303375487250225114210

**Published:** 2025-04-16

**Authors:** Yuting Guan, Zudong Xu, Liuling Liang, Wentao Deng, Zhilin Qin, Jiaming Yang, Shanyu Qin

**Affiliations:** 1 Department of Gastroenterology, The First Affiliated Hospital of Guangxi Medical University, Nanning, Guangxi, China;; 2 First Clinical Medical College, Guangxi Medical University, Nanning, Guangxi, China

**Keywords:** Acute pancreatitis, neutrophil, inflammation, hub genes, machine learning, biological functions, immune cells

## Abstract

**Background:**

Acute pancreatitis (AP) is a disease with high morbidity and mortality. Neutrophils are highly correlated with the occurrence and severity of the inflammatory response in AP. However, the mechanism by which neutrophils act on AP is currently unclear. We aimed to target genes coexpressed with neutrophils to identify new possibilities for diagnosing and treating AP.

**Methods:**

We analyzed the differences in immune infiltration in AP and the differential expression of neutrophil-coexpressed genes. Then, models were built through various machine learning methods, and the hub neutrophil coexpressed genes were identified. Receiver operating characteristic (ROC) curves were used for validation, and the relationships between the hub neutrophil coexpressed genes and immune infiltration were explored. GSEA and GSVA were used to investigate the biological functions of genes. Finally, we explored the miRNAs and lncRNAs associated with the hub neutrophil coexpressed genes.

**Results:**

Neutrophil infiltration varied significantly in samples from patients with AP. *RGS2*, *AHNAK*, and *OGT* were identified as hub neutrophil coexpressed genes by taking intersections of genes involved in the machine learning approach. The hub neutrophil coexpressed genes are closely associated with a variety of immune cells. GSVA revealed that the hub neutrophil coexpressed genes are differentially expressed in multiple immune- and inflammation-related pathways. These findings suggest that these genes may influence AP progression through these pathways.

**Conclusions:**

In this study, genes coexpressed with neutrophils in AP were identified, and their biological functions were investigated. These findings may provide more effective therapeutic strategies for the prediction, prevention, and personalized treatment of patients with AP.

## 1 INTRODUCTION

Acute pancreatitis (AP), an inflammatory pancreatic condition, is among the most intricate and clinically demanding abdominal diseases [1]. Gallstone migration and alcoholism are primarily responsible for AP. Additional causes are rare. The key characteristics of this illness include improper stimulation with trypsinogen, invasion of inflammatory cells, and breakdown of secretory cells. This illness is characterized by both localized and widespread inflammatory reactions, resulting in diverse clinical trajectories [2]. The 2012 Atlanta classification update categorizes AP into mild acute pancreatitis (MAP), moderately severe acute pancreatitis (MSAP), and severe acute pancreatitis (SAP) [3]. Currently, the main treatment strategies for AP involve sufficient resuscitation. In particular, early fluid resuscitation should be provided during the initial visit, and supportive care should be provided if the patient develops local or systemic complications [4]. Most patients exhibit self-resolving MAP, which typically diminishes within 1 week. Approximately one-fifth of patients experience moderate to severe acute pancreatitis, characterized by pancreatic necrosis, failure of peripancreatic tissue or organs, or both, with a mortality rate of 20–40% [5]. Although there has been a pathophysiological improvement in the understanding of the mechanisms and course of SAP in recent years [6, 7], a designated treatment for AP is lacking. Consequently, it remains essential to delve deeper into the potential origins of AP and to advance research focused on its treatment [8].

The inflammatory response is the underlying cause of the clinical manifestations of AP. Therefore, the key to disease treatment is to rationally modulate the mechanisms of the immune response, which in turn provides more effective therapeutic options [9]. The course and severity of AP are influenced by the interaction between innate and adaptive immune responses. Some studies revealed significant enrichment of certain susceptibility genes in biological processes associated with the immune response. This highlights the critical role of immune cells in the pathogenesis of AP [10]. The latest research indicates that neutrophils effectively limit the progression of infection through phagocytosis, degranulation, and neutrophil extracellular trap (NET) production to accomplish the clearance of pathogens and cellular fragments [11]. NETs are extracellular structures released by neutrophils that are involved in innate immunity that trap and kill pathogens to prevent their spread. In the context of AP, NETs have both benefits and limitations. Excessive release or aberrant function of NETs exacerbates the inflammatory response, leading to tissue damage and organ dysfunction [12]. Overproduction of NETs is associated with multiorgan dysfunction, thrombosis, and high mortality in AP patients. Therefore, targeting NETs or their components may be a new strategy for the treatment of SAP [13, 14]. However, the role of neutrophils is twofold. The lack of local inflammation induced by neutrophils in the later stages has the potential to cause systemic inflammation [15-17]. The role of neutrophils extends beyond merely combating pathogens and encompasses various physiological and pathological functions [18-21]. In general, neutrophils are crucial in the context of AP, with their role and control being important for comprehending and treating this disease. However, current studies have not elucidated the specific mechanism of neutrophil action in AP.

By performing gene coexpression pattern analysis of neutrophil infiltration in AP, this study highlights the critical role of genetic analysis in understanding immune mechanisms. Given that the enrichment analysis and single-gene analysis of neutrophil-coexpressed genes in AP were not performed by previous authors, we employed machine learning methods to deeply my neutrophil-coexpressed genes to gain an understanding of their potential functions and regulatory networks in the pathogenesis of AP. Comprehensive analysis revealed that neutrophil-coexpressed genes in AP may be critical for controlling neutrophil activation, the inflammatory response, and immune control in AP. This information is crucial for a deeper understanding of the pathogenesis of AP, the discovery of new therapeutic targets, and the development of more effective therapeutic strategies.

## 2 METHODS AND MATERIALS

### 2.1 Data Access and Processing

Access to the GSE194331 dataset was sourced from the GEO database (http://www.ncbi.nlm.nih.gov/geo/). The sample included RNA-Seq analysis of peripheral blood from 87 patients of AP and 32 healthy individuals [22]. Perl software was used to add annotation information to the platform file so that the probe matrix could transform into gene expression data.

### 2.2 Immune Infiltration Analysis

We used the CIBERSORT algorithm to estimate the relative abundance of different immune cell types in the samples [23]. The “barplot” package created bar graphs showing the relative percentages of different immune cell types in the control and treatment groups. The Pearson correlation test examined the relationship between 22 immune cells. The “ggplot2” package was used to plot box plots to show the distribution of expression among different immune cell types in different groups (control and treatment), and significance was marked.

### 2.3 Acquisition and Functional Enrichment Analysis of Neutrophil Coexpressed Genes

Pearson correlation analysis identified genes with a correlation coefficient > 0.4 associated with neutrophil expression levels [24, 25]. Enrichment analyses of Gene Ontology (GO) analyzed the roles of neutrophil-coexpressed genes [26]. At the same time, the Kyoto Encyclopedia of Genes and Genomes (KEGG) explored the roles of genes in multiple fields [27]. We carried out GO and KEGG based on the “clusterProfiler”, “org.Hs.eg.db”, “GOplot”, and “enrichplot” packages in R to verify whether we screened the genes for representativeness [28].

### 2.4 Differential Expression Analysis

Differentially expressed genes (DEGs) were analyzed and filtered from normal and AP samples in the datasets using the “limma” package in R. The filtering criteria were set to |logFC| > 0.5, adjusted *P*-values < 0.05 [10].

### 2.5 Searching for Hub Neutrophil Coexpressed Genes

We first performed an intersection analysis of DEGs and neutrophil-coexpressed genes and screened differentially expressed neutrophil-coexpressed genes. To further screen the hub neutrophil coexpressed genes, we applied the least absolute shrinkage and selection operator (LASSO) regression, random forest (RF), and support vector machine (SVM). The LASSO data mining tool introduces a penalty function in the commonly used multiple linear regression. It compresses the coefficients and streamlines the model, thus avoiding covariance and overfitting [29, 30]. RF employs decision trees to evaluate the significance of variables by assigning a value to each one [31]. The SVM is based on nonlinear mapping theory to find the optimal hyperplane of feature space division to help find the key samples and eliminate the redundant samples [32, 33]. Then, we identified the hub neutrophil coexpressed genes by taking intersections of the genes extracted by machine learning.

### 2.6 Exploration of Biological Functions of Hub Neutrophil Coexpressed Genes in AP

Based on the expression levels of hub neutrophil coexpressed genes, the samples were divided into high and low-expression groups to observe the differential expression of genes. Subsequently, gene set enrichment analysis (GSEA) analyses were performed on these differentially expressed genes using the “ClusterProfiler” package [34, 35]. Gene set variation analysis (GSVA) is a non-parametric, unsupervised technique for assessing transcriptome gene set enrichment [36]. We employed gene sets from the MSigDB database (https://www.gsea-msigdb.org/gsea/msigdb) and used the “GSVA” package to score each gene set comprehensively to assess potential changes in biological function across samples [37].

### 2.7 Single-Sample Genome Enrichment Analysis (ssGSEA)

We also used the “GSVA” package to perform ssGSEA on the AP genotyping results and then ranked and summarized the gene expression levels in the samples [38]. Moreover, we conducted a significant difference analysis and correlation analysis on the immune cell infiltration analysis results.

### 2.8 Construction of Competitive Endogenous RNA (ceRNA) Network

Firstly, Miranda [39], miRDB [40], and Targetscan [41] were used to predict the corresponding miRNAs of the hub neutrophil coexpressed genes. The intersection of the miRNAs predicted by the three databases was identified as the corresponding miRNAs of the genes. Then, the lncRNAs corresponding to these miRNAs were found using the spongeScan database (http://spongescan.rc.ufl.edu/) [42]. Cytoscape 3.10.2 was used to visualize and display the network diagrams of the genes, miRNAs, and lncRNAs [43].

### 2.9 Statistical Analysis

Perl software (version 5.18.2) was used to organize the data, and R software (version 4.1.2) was used to analyze the data. The Wilcoxon rank sum test or Student's t-test was implemented to determine whether were any significant differences between the two independent groups. Unless otherwise indicated, * P < 0.05 * was commonly regarded as statistically significant.

## 3 RESULTS

### 3.1 Landscape of Immune Infiltration in AP and Acquisition of Neutrophil Coexpressed Genes

**Fig. (1):**
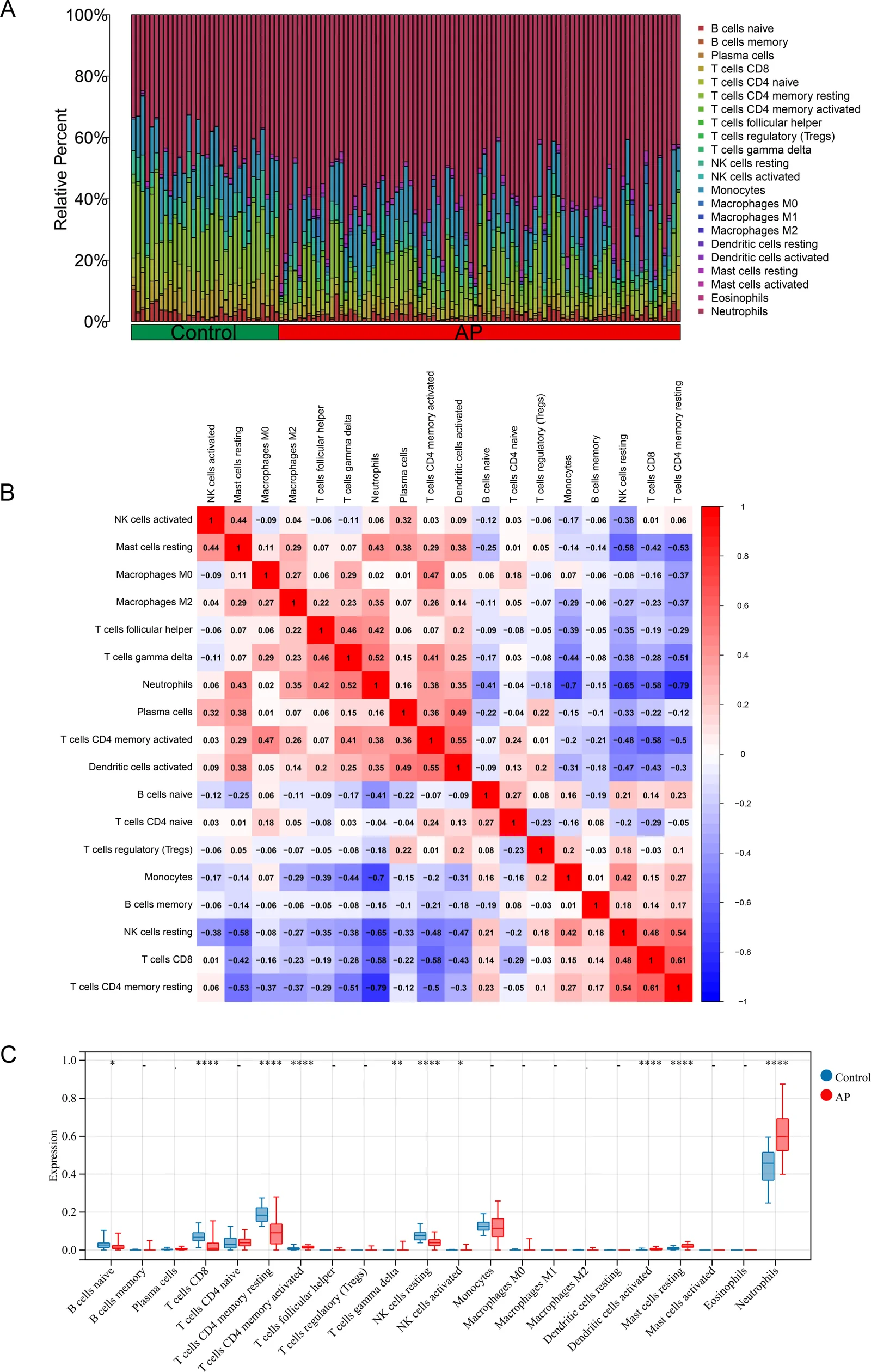
Landscape of immune infiltration in AP. (**A**) Bar plot of the composition of different immune cells in each AP and control samples. (**B**) Correlation analysis between immune infiltrating cells. Positive correlations are indicated in red, and negative correlations in blue. (**C**) The bar plot of various immune cell proportions in the AP and control groups. *P < 0.05; **P < 0.01; ***P < 0.001, ****P < 0.0001.

The immune cell infiltration obtained by the CIBERSORT algorithm showed that when comparing the disease and control groups, we observed significant differences in the infiltration of CD8+ T cells, T cells CD4+ memory resting, NK cells resting, Dendritic cells activated, Mast cells resting, Dendritic cells activated, Mast cells resting and neutrophils (Figs. **1A** and **1C**). In the present study, we observed correlations between different immune cell subpopulations. Specifically, there was a negative correlation between monocytes and neutrophils, NK cells resting and mast cells resting, NK cells resting and neutrophils, and CD8+ T cells and neutrophils (Fig. **1B**).

**Fig. (2):**
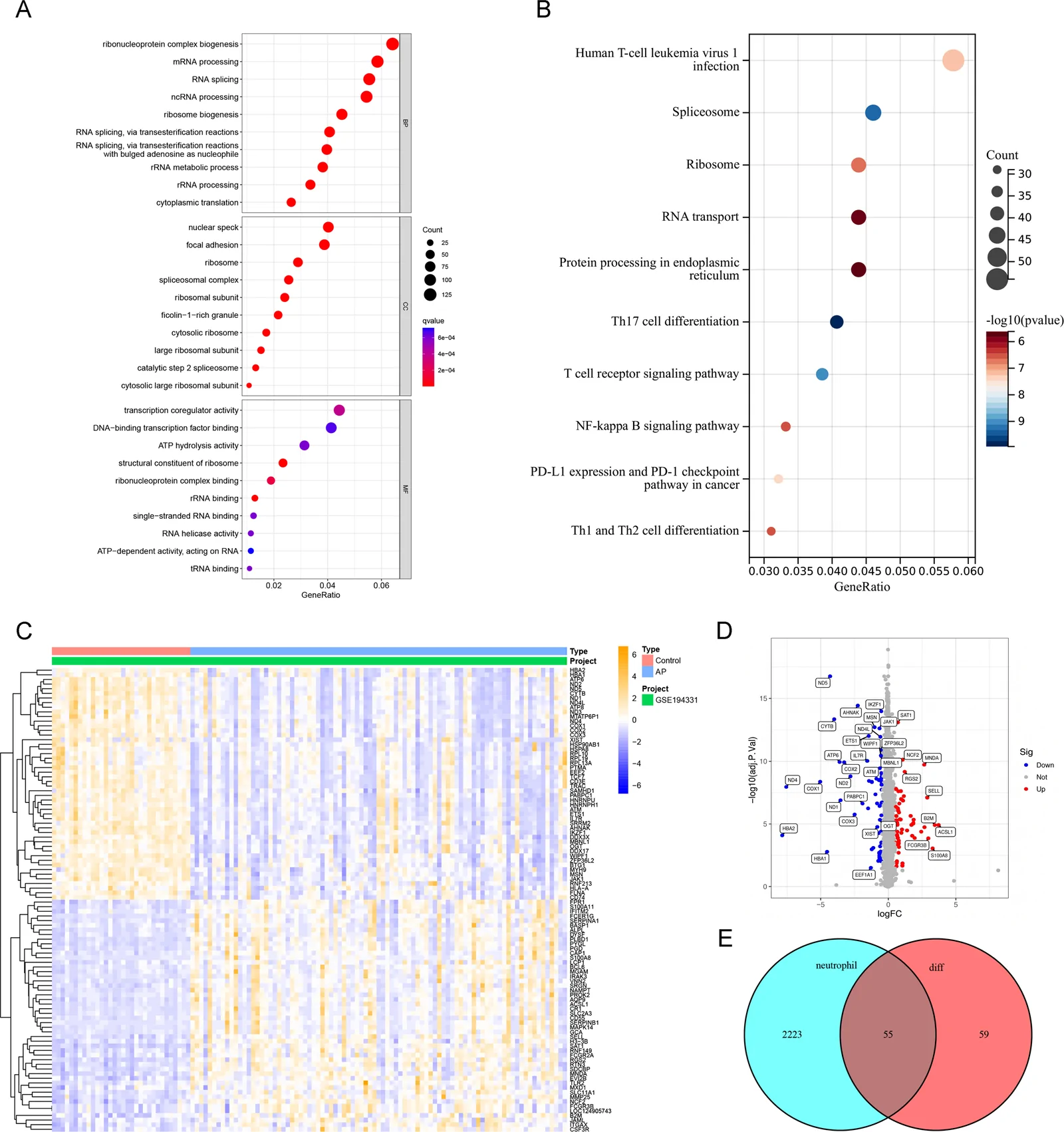
Screening for neutrophil and AP coexpressed genes. (**A**) A bar plot of GO analyses was performed on 2278 neutrophil coexpressed genes. (**B**) A bar plot of KEGG analyses was performed on 2278 neutrophil coexpressed genes. (**C**) Heat map of differentially expressed genes in AP and control groups. (**D**) The volcano plot reveals the DEGs between the control and AP groups. (**E**) The 55 coexpressed genes were obtained by taking the intersection of neutrophil coexpressed genes and DEGs.

The Pearson correlation analysis shows the interaction network of neutrophils with multiple genes and 2278 genes with a correlation coefficient > 0.4 associated with the expression levels of neutrophils were identified (Supplementary Table **1**). In GO analysis, neutrophil coexpressed genes were significantly related to biological processes, cellular components, and molecular functions such as ribonucleoprotein complex biogenesis, nuclear speckle, and transcription coregulator activity (Fig. **2A**). In KEGG analysis, neutrophil coexpressed genes were associated with Th17 cell differentiation, T cell receptor signaling pathway, NF-kappa B signaling pathway, and other pathways (Fig. **2B**). 

### 3.2 Differential Expression Analysis

Fig. (**2C**) shows the 50 genes with the most significant upregulation and downregulation. The results of differential expression analysis revealed 114 DEGs in the GSE194331 dataset, including 55 up-regulated genes and 59 down-regulated genes, which were altered in their expression levels in the AP group compared with the control group (Fig. **2D**).

### 3.3 Searching for Differential Neutrophil Coexpressed Genes in AP

The intersection result of the 2278 neutrophil coexpressed genes, and 114 DEGs was visualized as a Venn diagram, resulting in 55 differential neutrophil coexpressed genes for further study (Fig. **2E**, Supplementary Table **2**).

### 3.4 Identification of Hub Neutrophil Coexpressed Genes

**Fig. (3):**
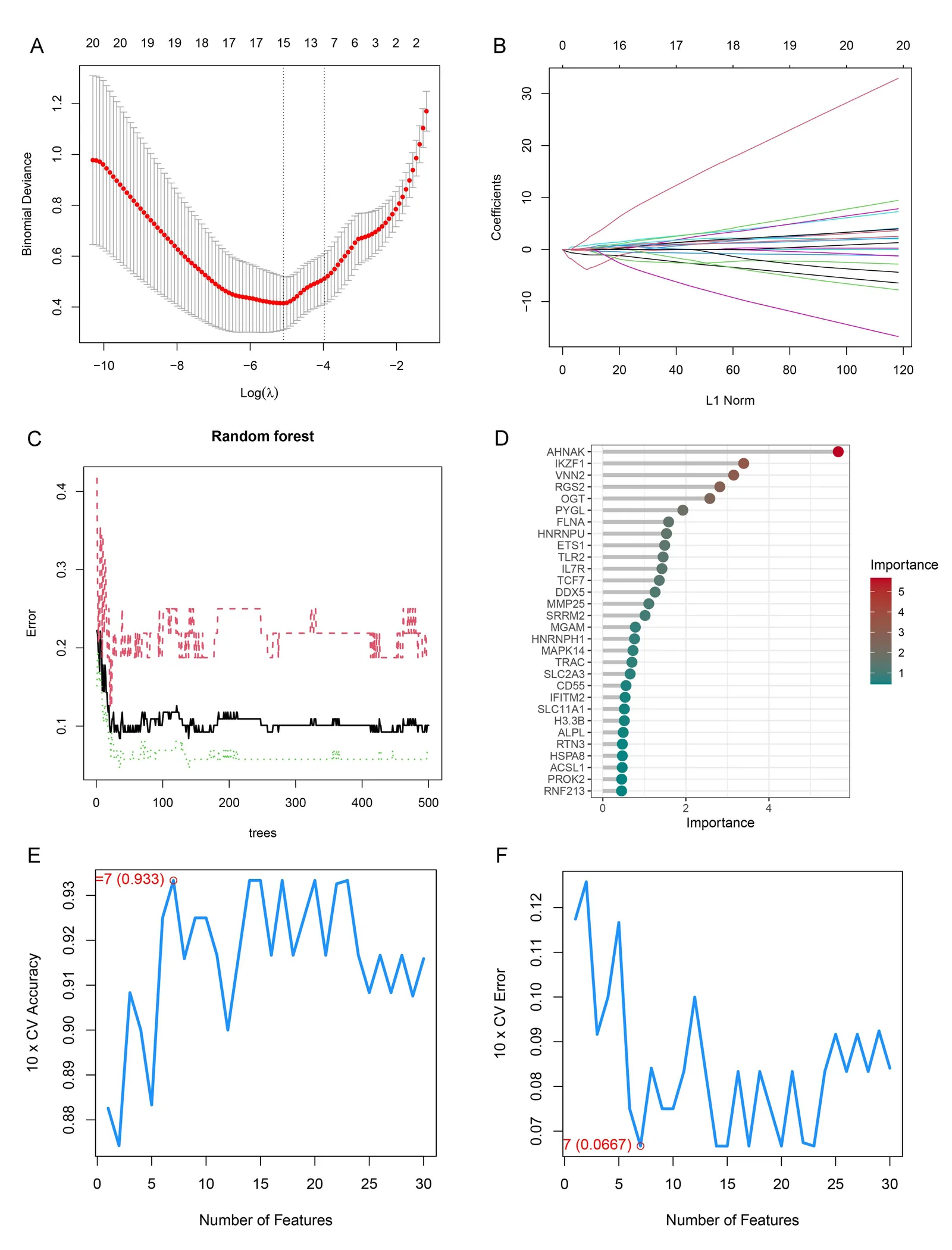
Screening for hub neutrophil coexpressed genes. (**A**) Coefficient spectrum in the LASSO regression model. (**B**) LASSO regression analysis cross-validation curves indicating the convergence screening process. (**C**) Reverse cumulative distribution of residual indicates the error rates when selecting different numbers of trees. (**D**) Importance scores for each gene. (**E, F**) Graphs of accuracy and error of cross-validation. The horizontal coordinates represent the number of genes. The vertical coordinate of the left graph represents the accuracy of cross-validation, and the vertical coordinate of the right graph represents the error of cross-validation.

We performed an intersection analysis of DEGs and neutrophil-coexpressed genes and finally identified 55 differential neutrophil-coexpressed genes. The LASSO regression algorithm was used for characterization and selection, and finally, 15 genes were extracted (Figs. **3A** and **B**). Meanwhile, 5 genes were screened by the RF algorithm (Figs. **3C** and **D**). Finally, 7 genes were screened by the SVM algorithm (Fig. **3E** and **F**).

**Fig. (4):**
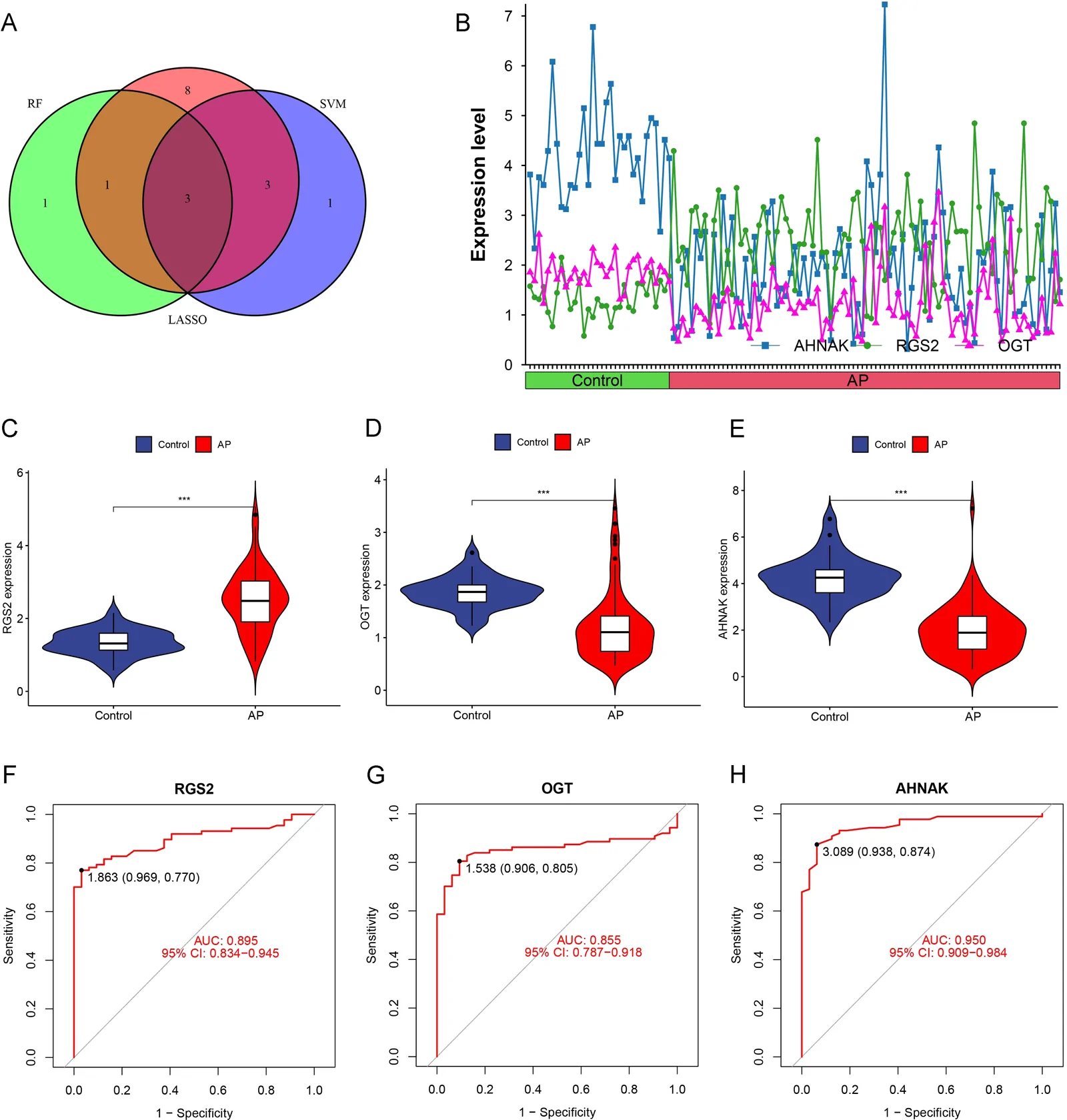
Identification and validation of hub neutrophil coexpressed genes. (**A**) The hub neutrophil coexpressed genes were obtained by taking the intersection of the genes extracted by LASSO, RF, and SVM. (**B**) The expression level of RGS2, OGT, and AHNAK in AP. (**C**) The expression levels of RGS2 in GSE194331. (**D**) The expression levels of OGT in GSE194331. (**E**) The expression levels of AHNAK in GSE194331. (**F**) The ROC curve of the diagnostic efficiency of RGS2 in the GSE194331. (**G**) The ROC curve of the diagnostic efficiency of OGT in the GSE194331. (**H**) The ROC curve of the diagnostic efficiency of AHNAK in the GSE194331. *P < 0.05; **P < 0.01; ***P < 0.001.

By taking the intersection of the genes extracted by the methods of LASSO regression, RF, and SVM, we obtained the hub neutrophil coexpressed genes: *RGS2*, *OGT*, and *AHNAK*. The genes are critical for neutrophil regulation in AP (Fig. **4A**). The expression levels of the genes in different samples are shown in detail in (Fig. **4B**). *RGS2* was highly expressed in samples of AP (Fig. **4C**), and *OGT* and *AHNAK* were lowly expressed in AP (Figs. **4D** and **E**). The diagnostic ability of the genes in AP was further validated in Figs. (**4F**-**H**), where they all had good diagnostic ability (all AUC > 0.85).

### 3.5 Exploration of Biological Functions of Hub Neutrophil Coexpressed Genes in AP

**Fig. (5):**
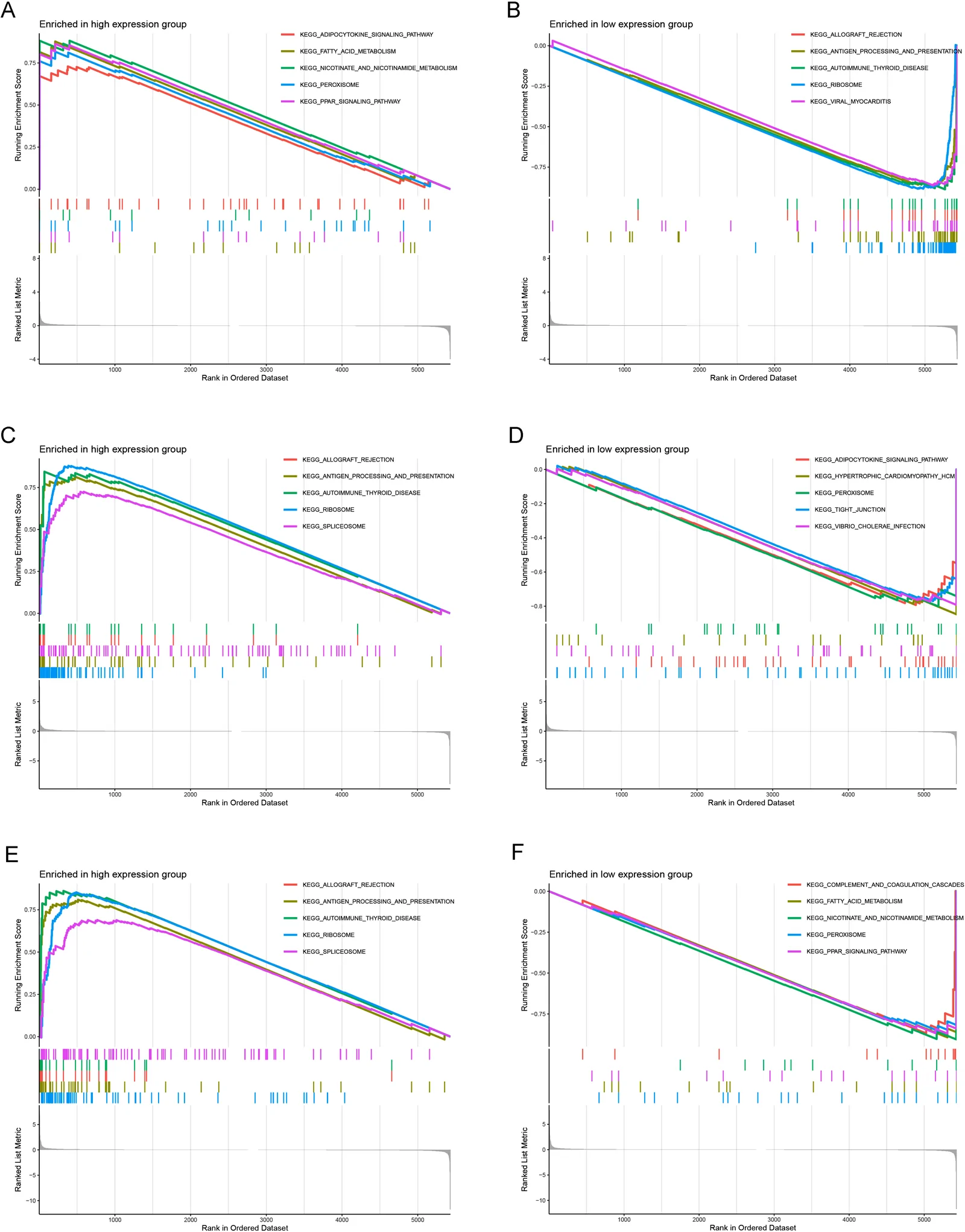
GSEA functional analysis of hub neutrophil coexpressed genes. (**A**) Results of GSEA analysis of the high expression group of RGS2. (**B**) Results of GSEA analysis of the low expression group of RGS2. (**C**) Results of GSEA analysis of the high expression group of OGT. (**D**) Results of GSEA analysis of the low expression group of OGT. (**E**) Results of GSEA analysis of the high expression group of AHNAK. (**F**) Results of GSEA analysis of the low expression group of AHNAK.

**Fig. (6):**
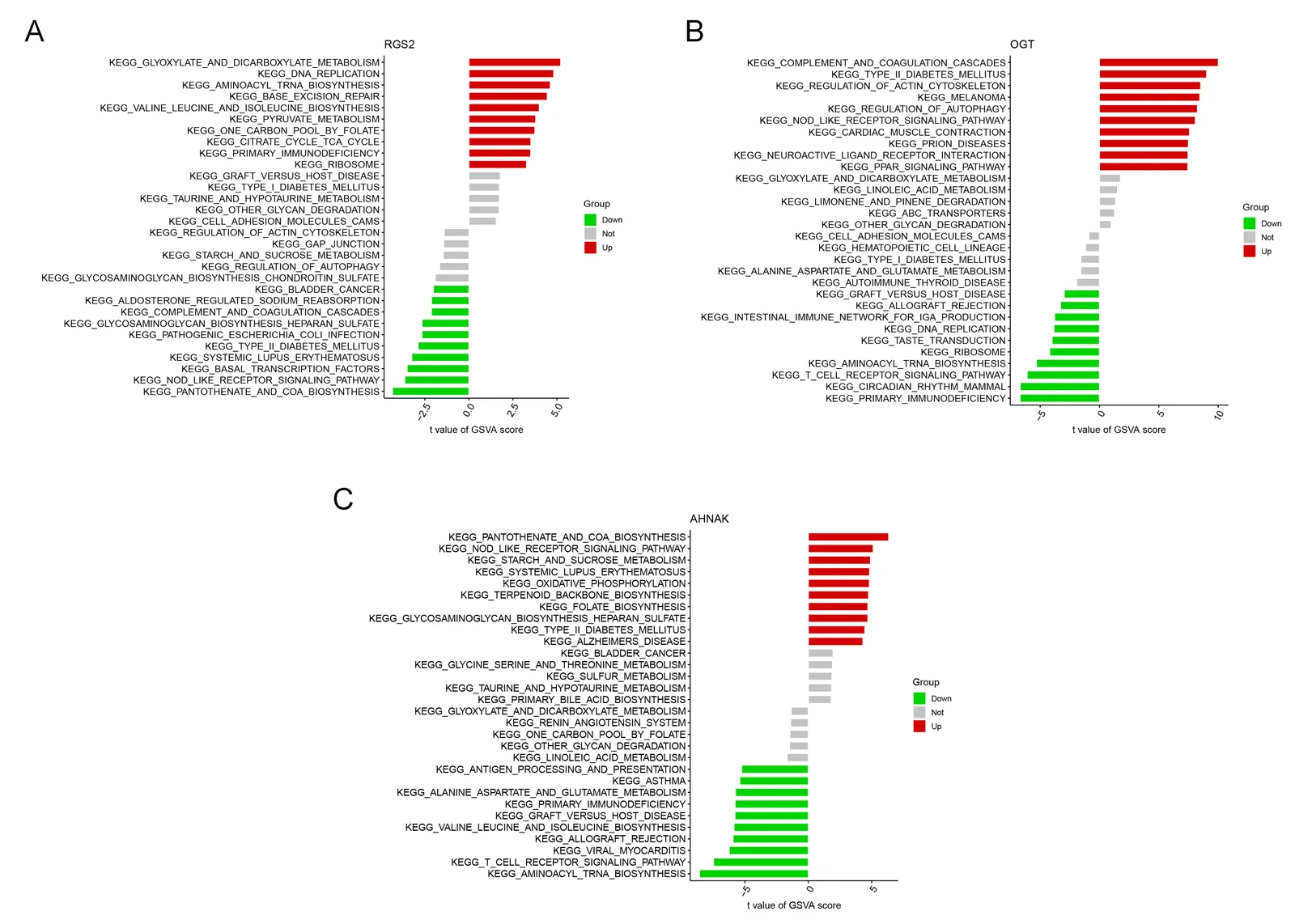
GSVA functional analysis of hub neutrophil coexpressed genes. (**A**) GSVA analysis of RGS2 demonstrated enrichment of immune-related KEGG pathways. (**B**) GSVA analysis of OGT demonstrated enrichment of immune-related KEGG pathways. (**C**) Results of GSVA analysis of AHNAK.

The samples were categorized into groups of high and low expressions, depending on the gene expression levels. The GSEA analysis of genes revealed that *RGS2*, *OGT*, and *AHNAK* were significantly enriched in different pathways in AP (Fig. **5**). It provides a conjecture to discover the mechanism of gene action in AP. To better explain the biological phenomena, we further employed GSVA analysis. The GSVA results of *RGS2* show that the set of highly expressed genes is involved in key biological processes such as protein synthesis, immune system activity, energy metabolism, DNA synthesis, and repair. In contrast, its low-expression gene set includes cofactor synthesis, immune signaling, basic transcription factors, and pathways associated with autoimmune disease and cancer (Fig. **6A**). According to the GSVA results of *OGT*, we can observe biological processes related to high and low expression gene sets, such as primary immunodeficiency, autophagy regulation, intestinal immune IgA production, type 2 diabetes, and complement and coagulation cascade reactions (Fig. **6B**). Finally, the GSVA results of *AHNAK* show that the highly expressed gene set is involved in biological processes, such as Alzheimer's disease, type 2 diabetes mellitus, and folate biosynthesis. In contrast, the low-expression gene set includes biological processes such as primary immunodeficiencies, asthma, and antigen processing and presentation (Fig. **6C**).

### 3.6 Immune Infiltration Analysis of Hub Neutrophil Coexpressed Genes

**Fig. (7):**
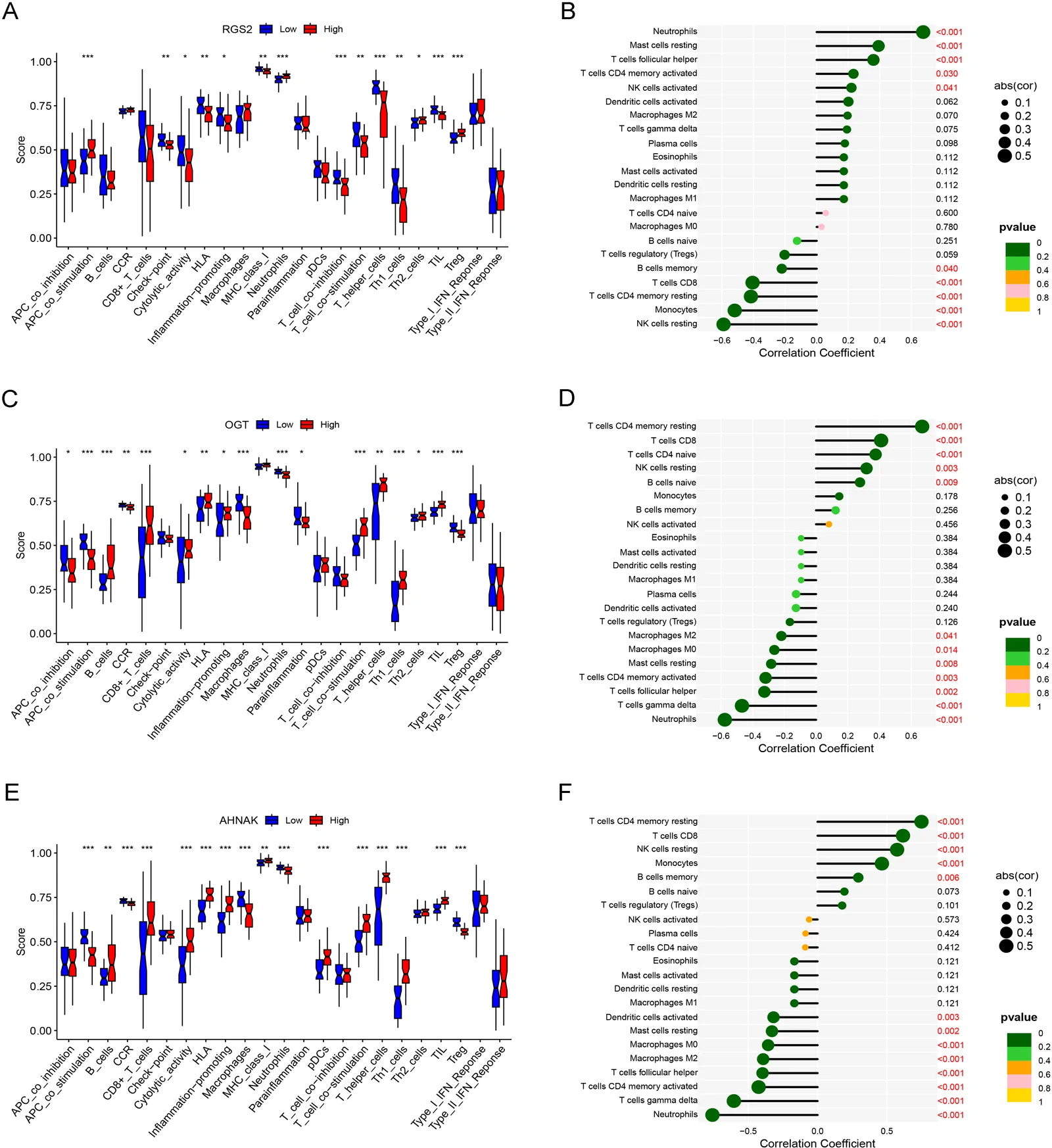
Immune infiltration analysis of hub neutrophil coexpressed genes. (**A**) The immune cell levels of RGS2 high and low expression groups. (**B**) Correlation between RGS2 and infiltrating immune cells. (**C**) The immune cell levels of OGT high and low expression groups. (**D**) Correlation between OGT and infiltrating immune cells. (**E**) The immune cell levels of AHNAK high and low expression groups. (**F**) Correlation between AHNAK and infiltrating immune cells. *P < 0.05; **P < 0.01; ***P < 0.001.

The hub neutrophil coexpressed genes were differentially expressed in immune-infiltrating cells (Figs. **7**, **7A**, **C** and **E**). To assess the correlation between *RSG2*, *OGT*, and *AHNAK* and immune infiltrating cells. *RGS2* was positively correlated with neutrophils, mast cells resting, and T cells follicular helper, and negatively correlated with NK cells resting and monocytes, among others. *OGT* was positively correlated with T cells CD4+ memory resting, CD8+ T cells, and T cells naïve and negatively correlated with neutrophils and T cells gamma delta. *AHNAK* was positively correlated with T cells CD4+ memory resting, CD8+ T cells, NK cells resting, and monocytes, and negatively correlated with neutrophils and T cells gamma delta, among others (Figs. **7B**, **7D** and **7F**).

### 3.7 Construction of ceRNA Network

**Fig. (8):**
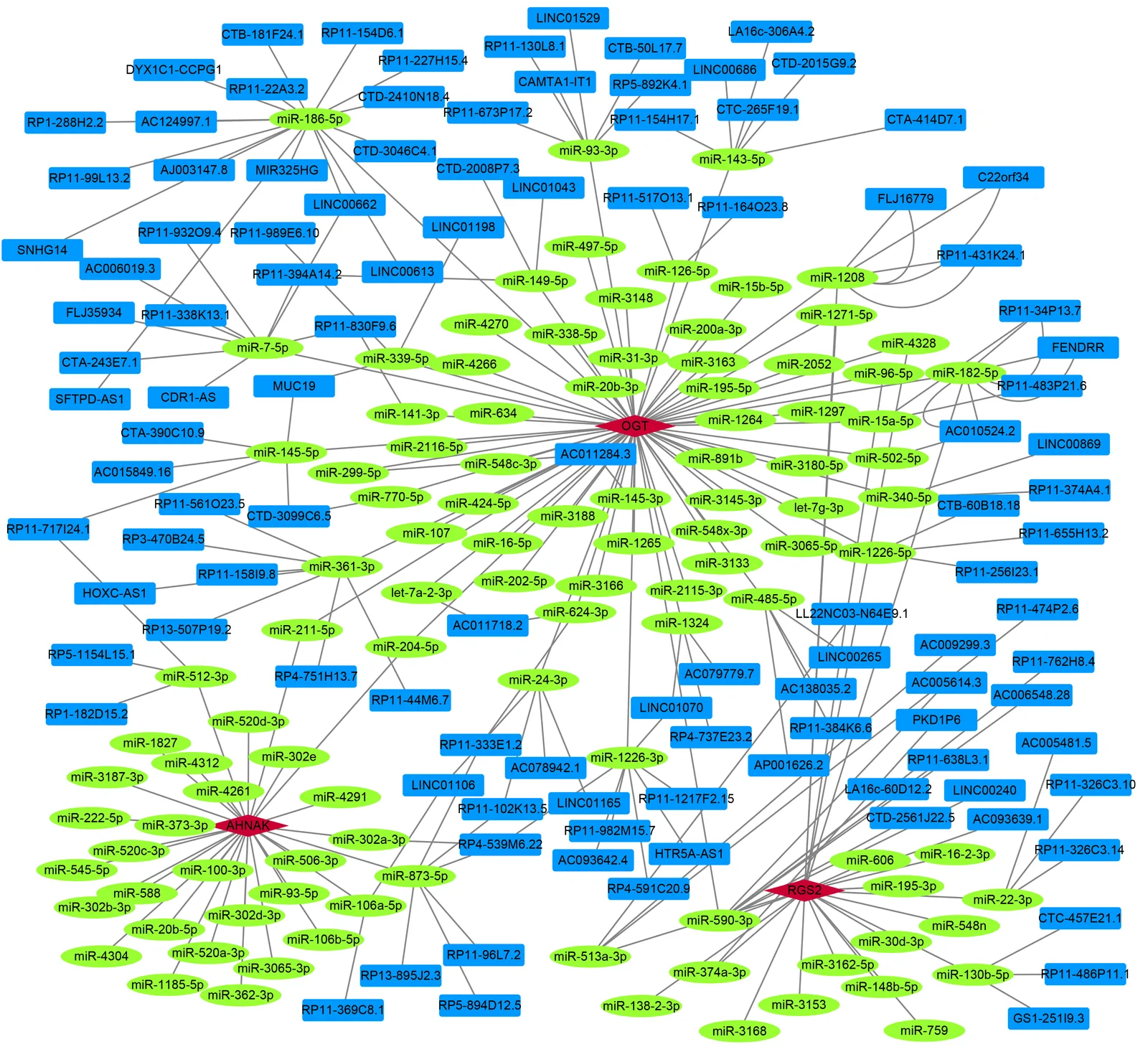
Construction of ceRNA networks associated with hub neutrophil coexpressed genes. The red diamond-shaped square represents the hub neutrophil coexpressed genes, the green circle represents the miRNAs that target and regulate hub neutrophil coexpressed genes, and the blue square frame represents the lncRNAs that target miRNAs.

In this study, we found that predicting miRNAs and lnRNAs in hub neutrophil coexpressed genes associated with AP involves several important regulatory factors (Fig. **8**). Table **1** shows the ceRNA network of hub neutrophil coexpressed genes, which have robust regulatory relationships between these molecules. For *OGT*, 26 miRNAs and 104 lncRNAs may be involved in the regulation process. For *RGS2*, we detected 6 miRNAs and 16 lncRNAs. For *AHNAK*, we speculated that 4 miRNAs and 12 lncRNAs may be its potential regulators (Table **1**). These findings provide useful clues for further investigating these genes and their regulatory networks.

## 4 DISCUSSION

AP is one of the most common gastrointestinal disorders requiring emergency hospitalization [44], especially in high-income countries, where it has an annual incidence of 34/100,000 person-years [45]. Acute pancreatitis (AP) is usually triggered by excessive alcohol consumption, a high-fat diet, or gallstones. Initial pancreatic injury results in altered intestinal permeability, which allows intestinal microorganisms to enter the pancreas [46-48]. Intestinal bacteria invade pancreatic tissue and subsequently spread the pathogen to distant organs. Most patients experience MAP, which usually resolves spontaneously within 1 week. However, approximately 20% of patients develop moderate or severe acute pancreatitis, which may be associated with organ necrosis and has a mortality rate of 20–40% [49-51]. Even those who survive may experience long-term consequences that severely impact their quality of life, including diabetes, pancreatic exocrine insufficiency, and chronic pancreatitis [52]. Venkatesh *et al.* [53] reported that early pancreatic tissue injury triggered by AP activates the innate immune response, leading to an inflammatory cascade associated with AP. A variety of immune cells are involved in the inflammatory response process in AP, including neutrophils. Neutrophil recruitment, granulocyte release, and NET formation are closely associated with AP exacerbation. Pharmacological inhibition of neutrophil behavior or product production has become a therapeutic strategy for AP [54, 55]. Currently, there is no specific pharmacologic therapy for AP. The treatment of AP is based on initial therapy, including assessment of disease severity, fluid resuscitation, pain control, nutritional support, antibiotic use, and endoscopic retrograde cholangiopancreatography (ERCP) therapy, among others [56]. Therefore, this study aims to provide new insights for precision therapy for AP by identifying new biological targets associated with neutrophil and AP coexpression and exploring their potential mechanisms of action.

We analyzed the RNA-seq data of samples from patients with AP for immune cell infiltration *via* the CIBERSORT algorithm. When we compared the disease and control groups, we observed CD8+ T cells, quiescent CD4+ memory T cells, quiescent NK cells, activated dendritic cells, quiescent mast cells, and neutrophils. There were significant differences in the amount of infiltration. Ultimately, we chose neutrophil marker genes as a candidate gene set for the prediction model. By constructing a neutrophil coexpression network in AP and screening it in multiple ways, we identified three hub neutrophil coexpressed genes of the coexpression network.

RGS2 is an RGS protein involved in G protein-coupled receptor (GPCR) signaling. It plays a key role in physiology and disease, particularly in the cardiovascular system, where it has multiple functions, including blood pressure regulation. The protein stops signaling by promoting hydrolysis of GTP by the Gα subunit and is a regulator of GTPase-activating protein (GAP) activity [57]. The high expression of RGS2 in cell growth, tissue remodeling, and exosomal vesicles suggests that RGS2 may be involved in immune regulation and cell signaling [58]. George *et al.* reported that Rgs2 knockout mice (Rgs2-/-) presented bronchoprotective effects after acute neutrophilic inflammation, particularly exposure to lipopolysaccharide (LPS), whereas Rgs2-/- mice presented more severe airway hyperresponsiveness (AHR) and abnormal lung function [59]. In an acute inflammation model, Rgs2 knockdown resulted in a significant increase in neutrophil inflammation and IL12B gene expression, emphasizing the multiple roles of RGS2 in inflammation and immune responses [59]. The RGS2 gene may regulate neutrophil infiltration through multiple pathways in AP. The GSVA analysis of RGS2 in this study revealed that the RGS2 gene is involved in regulating several biological processes, including the immune system, cardiovascular system, and infectious diseases. It plays a key role in antigen processing, presentation, and graft rejection.

O-GlcNAc transferase (OGT) genes are genes that encode O-GlcNAc glycosyltransferases, which play key regulatory roles in the cell [60, 61]. OGT plays a multifaceted role in the immune system and inflammation through O-GlcNAcylation. In the nuclear factor kappa-B (NF-κB) signaling pathway, OGT enhances the functional activity of several key molecules in the TLR-NF-κB signaling pathway through O-GlcNAcylation and promotes the transcription of NF-κB target genes, thereby activating innate immune cells and triggering inflammatory responses [62]. In addition, the upregulation of OGT decreased STAT3-IL-10 signaling in myeloid cells and enhanced the inflammatory response and disease severity in patients with colitis [63]. Research has shown that the absence of OGT may lead to the activation of RIPK3, increasing inflammatory vesicle activation and IL-1β production and further exacerbating the inflammatory response. However, OGT-mediated O-GlcNAc signaling also inhibits innate immune activation and necroptosis through O-GlcNAcylation of RIPK3 apoptotic signaling, which is important for inhibiting inflammatory responses and cell death [64]. A reduction in OGT is known to attenuate the severity of Cerulein-induced AP inflammatory response in a mouse model [65]. The underlying mechanism may be related to OGT-mediated O-GlcNAcylation promoting NF-κB signaling activation and inflammation in pancreatic alveolar cells [66]. GSVA revealed that OGT genes are involved in multiple biological processes, such as primary immunodeficiency, intestinal immune IgA production, and T-cell receptor signaling pathways. These research results indicate that OGT plays different roles in various physiological and disease processes, and future in-depth studies will help reveal its specific mechanisms in disease development and immune regulation. Overall, these results provide new research directions for exploring the mechanism of action and therapeutic applications of O-GlcNAcylation in immunity and inflammation.

AHNAK is a multifunctional protein involved in a variety of biological processes, such as the inflammatory response, calcium channel regulation, blood-brain barrier formation, lipid metabolism, and membrane repair [67, 68]. AHNAK is involved in multiple aspects of pathophysiological mechanisms and is particularly closely related to NF-κB signaling and immune processes [69]. Its association with mutant beta-type platelet-derived growth factor receptors (PDGFRBs) activates extracellular signal-regulated kinase (ERK) and NF-κB signaling, triggering cell motility and the expression of inflammation-related genes, which may lead to aberrant cell behavior and inflammation [70]. In addition, the expression of AHNAK in inflammation-induced luminal structures has attracted attention [71]. Moreover, AHNAK inhibited multiple signaling pathways, including the Wnt/β-linker protein, ERK, and protein kinase B (AKT) signaling pathways, reducing epithelial-mesenchymal transition (EMT), migration, and invasion of tumor cells. AHNAK is abnormally highly expressed in pancreatic ductal adenocarcinoma (PDAC), which is associated with shorter disease-free survival and poorer overall survival. Knockdown of AHNAK reversed P53 downregulation and inhibited the EMT, proliferation, and migration of PDAC cells [72]. GSVA revealed that AHNAK expression is associated with immune regulatory functions. This comprehensive analysis highlights the potential biological processes in which AHNAK is involved at multiple levels, but the specific mechanisms still need to be validated by further in-depth studies. Research on neutrophil therapy strategies may provide more precise or targeted therapies for patients with AP that minimize the occurrence of systemic inflammation.

AP is a disease that severely affects the quality of life and immune function and neutrophil-coexpressed genes (*RGS2*, *AHNAK*, and *OGT*) may play key roles in the development of this disease. However, this study has several limitations. First, a single dataset may not be sufficient to fully explain the complexity of a biological system, as it may provide only limited information to cover all relevant factors. Integrating multiple datasets can increase the coverage and reliability of the analyses and help to obtain more comprehensive information [73]. Second, there was no experimental verification of gene expression in this study, which reduces the credibility of the findings. It is necessary to improve the relevant basic research to verify the reliability and biological significance of these analyses. Future research should focus on an in-depth exploration of the regulatory mechanisms of these genes, as well as increasing the sample size and diversity, to elucidate the comprehensive pathophysiological mechanisms of AP. Third, the associations between immune cells, especially neutrophils, and hub neutrophil coexpressed genes are based on expression and correlation analyses, and more reliable association studies are lacking. It is still unknown how these genes recruit, activate, and regulate immune cells to affect AP. In addition, although we constructed ceRNAs to attempt to explain potential regulatory mechanisms, further experimental validation is needed. Fourth, the current research is based on mRNA expression and excludes analyses of functional proteins and metabolites. In the future, it will be necessary to integrate information from different levels, such as regulatory factors, proteins, and metabolites to construct the regulatory network. This will enable a deeper understanding of the molecular mechanisms and genetic basis of complex traits in the biological and disease processes of AP [74]. Finally, due to the lack of comprehensive information on the clinical and severity of AP patients in complex clinical contexts in public databases, it is not possible to further validate the association between hub neutrophil coexpressed genes and the severity and mortality of AP. In addition, the recruitment and release of neutrophils, as well as the formation of NETs, are closely related to the deterioration of AP [55, 75]. By targeting central genes and implementing anti-neutrophil therapy in AP, it is possible to effectively limit neutrophil-mediated tissue damage, thereby minimizing pancreatic injury and systemic inflammatory response. However, future clinical studies are needed to further validate the feasibility and safety of these strategies.

## CONCLUSION

AP, characterized by pancreatic inflammation, significantly affects quality of life. This study identified hub neutrophil coexpressed genes (*RGS2*, *AHNAK*, and *OGT*) by analyzing the genes coexpressed with neutrophil infiltration in AP. Examination of the RNA-Seq data revealed a close relationship between the hub neutrophil coexpressed genes and infiltrating immune cells. Hub neutrophil coexpressed genes could be crucial in controlling neutrophil activation, inflammatory reactions, and immune control. These findings provide important insights for a deeper understanding of the pathophysiological mechanisms of AP and the development of therapeutic strategies.

## Figures and Tables

**Table 1:** CeRNA regulatory network of hub neutrophil coexpressed genes.

mRNA	miRNA	lncRNA
*RGS2*	miR-22-3p	RP11-326C3.10, RP11-326C3.14, AC005481.5
	miR-1208	FLJ16779, RP11-431K24.1, C22orf34
	miR-182-5p	RP11-34P13.7, FENDRR, AC010524.2
	miR-130b-5p	GS1-251I9.3, RP11-486P11.1, CTC-457E21.1
	miR-374a-3p	PKD1P6
	miR-513a-3p	RP11-474P2.6, LL22NC03-N64E9.1, AC009299.3
*OGT*	let-7a-2-3p	AC011718.2
	miR-1208	FLJ16779, RP11-431K24.1, C22orf34
	miR-1226-3p	LINC01070, RP4-539M6.22, RP11-1217F2.15, RP11-982M15.7, AC093642.4, RP4-591C20.9, HTR5A-AS1
	miR-1226-5p	CTB-60B18.18, RP11-256I23.1, RP11-655H13.2
	miR-126-5p	RP11-164O23.8, RP11-517O13.1
	miR-1324	AC079779.7, RP4-737E23.2, LINC01070
	miR-141-3p	RP11-830F9.6
	miR-143-5p	CTA-414D7.1, CTC-265F19.1, LA16c-306A4.2, LINC00686, RP11-154H17.1, CTD-2015G9.2
	miR-145-3p	AC011284.3
	miR-145-5p	MUC19, CTD-3099C6.5, CTA-390C10.9, RP11-717I24.1, AC015849.16
	miR-149-5p	LINC01043, RP11-394A14.2, CTD-2008P7.3
	miR-15a-5p	RP11-483P21.6, RP11-34P13.7
	miR-182-5p	RP11-34P13.7, FENDRR, AC010524.2
	miR-186-5p	RP11-99L13.2, MIR325HG, LINC00613, DYX1C1-CCPG1, RP11-154D6.1, RP11-22A3.2, SFTPD-AS1, RP1-288H2.2, AC124997.1, CTD-2410N18.4, AJ003147.8, CTD-3046C4.1, RP11-227H15.4, LINC00662, CTB-181F24.1, SNHG14
	miR-24-3p	RP11-102K13.5, LINC01106, LINC01165, AC078942.1
	miR-299-5p	AC011284.3
	miR-339-5p	MUC19, RP11-989E6.10, LINC01198
	miR-340-5p	LINC00869, RP11-374A4.1
	miR-361-3p	RP3-470B24.5, RP13-507P19.2, HOXC-AS1, RP11-158I9.8, RP11-44M6.7, RP11-561O23.5, RP4-751H13.7
	miR-485-5p	AP001626.2, LINC00265, RP11-384K6.6, AC138035.2
	miR-502-5p	AC010524.2
	miR-590-3p	LINC00240, AC005614.3, RP11-762H8.4, AC006548.28, CTD-2561J22.5, LA16c-60D12.2, RP11-638L3.1
	miR-624-3p	RP11-333E1.2, AC011718.2
	miR-7-5p	CDR1-AS, RP11-830F9.6, FLJ35934, RP11-338K13.1, RP11-932O9.4, AC006019.3, RP11-394A14.2, LINC00662, CTA-243E7.1
	miR-770-5p	CTD-3099C6.5
	miR-93-3p	RP5-892K4.1, RP11-130L8.1, CAMTA1-IT1, CTB-50L17.7, RP11-673P17.2, LINC01529
*AHNAK*	miR-106a-5p	LINC01106, RP11-369C8.1
	miR-302a-3p	RP4-539M6.22
	miR-512-3p	RP11-717I24.1, RP1-182D15.2, RP5-1154L15.1
	miR-873-5p	RP5-894D12.5, RP11-102K13.5, RP4-539M6.22, RP11-333E1.2, RP11-96L7.2, RP13-895J2.3

## Data Availability

The datasets supporting the conclusions of this study are included in the GEO database.

## LIST OF ABBREVIATIONS

MAP: Mild Acute Pancreatitis
ROC: Receiver Operating Characteristic
AP: Acute Pancreatitis
MSAP: Moderately Severe Acute Pancreatitis
SAP: Severe Acute Pancreatitis
