# Supplementary material for: Identification of Hub Neutrophil Coexpressed Genes in Acute Pancreatitis via Machine Learning
Source: Endocr Metab Immune Disord Drug Targets. 2025 Apr 16;26:E18715303375487. doi: 10.2174/0118715303375487250225114210 (PMC13598744; doi:10.2174/0118715303375487250225114210)
Supplement: Supplementary file 1 [file EMIDDT-26-E18715303375487_SD1.pdf]

Supplementary Material

Identification of Hub Neutrophil Coexpressed Genes in Acute Pancreatitis via Machine Learning

Yuting Guan[1], Zudong Xu[2], Liuling Liang[1], Wentao Deng[1], Zhilin Qin[1], Jiaming Yang[1] and Shanyu Qin[1,*]

[1]Department of Gastroenterology, The First Affiliated Hospital of Guangxi Medical University, Nanning, Guangxi, Chin; [2]First Clinical Medical College, Guangxi Medical University, Nanning, Guangxi, China

Supplementary Table 1. 2278 neutrophil coexpressed genes in acute pancreatitis.

Gene	Cor	P value
PPP4R1	0.699052208	1.10E-12
MYO7B	0.673385771	1.43E-11
TNFRSF10C	0.668521387	2.26E-11
NDEL1	0.66511969	3.10E-11
CXCR2	0.664296264	3.34E-11
ZNF608	0.66310059	3.73E-11
HECW2	0.656925353	6.51E-11
THBD	0.656672441	6.66E-11
MAK	0.656318365	6.88E-11
LINC02207	0.652452942	9.68E-11
PPM1D	0.652339131	9.77E-11
REPS2	0.650581384	1.14E-10
CCNJL	0.649590915	1.24E-10
TREM1	0.647845468	1.45E-10
TREML2	0.647689623	1.46E-10
IRAG1	0.64457877	1.91E-10
CXCR1	0.643610569	2.08E-10
LOC101928143	0.641674781	2.45E-10
LYVE1	0.637602903	3.44E-10
NRBF2	0.635356007	4.14E-10
RTN3	0.635034051	4.26E-10
RGS2	0.63436292	4.50E-10
OSBPL1A	0.632418215	5.27E-10
VPS37C	0.631254821	5.80E-10
CHRNA10	0.630963296	5.94E-10
PACSIN2	0.629559609	6.65E-10
UIMC1	0.628249145	7.39E-10
SLC15A4	0.625336689	9.33E-10
CMTM2	0.625007903	9.58E-10
SVIL	0.624181188	1.02E-09
CXCL16	0.623443793	1.08E-09

MAP2K4	0.6232794	1.10E-09
SLC5A9	0.620905941	1.32E-09
TECPR2	0.620792129	1.34E-09
AREL1	0.620454617	1.37E-09
ARHGEF40	0.619928299	1.43E-09
RNF24	0.61642164	1.88E-09
ARHGAP26	0.615925489	1.95E-09
H2BC4	0.61537197	2.03E-09
GLT1D1	0.614360323	2.20E-09
NFIL3	0.614132703	2.24E-09
LRRC4	0.613731925	2.31E-09
CEP19	0.613702753	2.31E-09
NUMB	0.61297013	2.44E-09
FAM53C	0.61244594	2.54E-09
LOC100128966	0.61244594	2.54E-09
EXTL3	0.612239736	2.58E-09
RAB36	0.612033536	2.62E-09
HNRNPA2B1	-0.611681242	2.69E-09
ZFAS1	0.611658036	2.70E-09
CFAP58	0.607481126	3.70E-09
CFAP92	0.607282637	3.75E-09
M6PR	-0.606641544	3.94E-09
STS	0.604774972	4.52E-09
KIF13A	0.60472439	4.54E-09
SLC8A1	0.603653332	4.91E-09
GTF2IP4	0.603105755	5.11E-09
DAZAP1	-0.60246464	5.36E-09
BAZ2B-AS1	0.59974203	6.54E-09
SIRPB2	0.599028807	6.89E-09
SUPT7L	-0.598974437	6.91E-09
IRF2BPL	0.598730384	7.04E-09
SCN1B	0.598696232	7.06E-09
ECE1	0.596887903	8.04E-09
PHLPP1	0.596757673	8.12E-09
CPQ	0.596571761	8.23E-09
MAML3	0.596175976	8.46E-09
HTATSF1	-0.590384299	1.28E-08
N4BP1	0.589792228	1.33E-08
DGAT2	0.589625564	1.35E-08
RFX2	0.589549691	1.36E-08
LOC124900275	0.589473817	1.36E-08
SLC31A2	0.587631278	1.55E-08
BCL2L11	0.587492176	1.57E-08
NIBAN1	0.587251005	1.59E-08

FCGR3B	0.586658439	1.66E-08
LOC124905743	0.586658439	1.66E-08
HAL	0.586458939	1.68E-08
CRACDL	0.586287131	1.70E-08
CDKL5	0.585604269	1.78E-08
DNTTIP1	0.585427231	1.81E-08
GAB2	0.585147482	1.84E-08
PYGM	0.584896117	1.87E-08
LYN	0.584755924	1.89E-08
SERBP1	-0.584061508	1.99E-08
CHST15	0.583796499	2.02E-08
OSBPL2	0.58357202	2.05E-08
LOC105369209	0.58357202	2.05E-08
REM2	0.583176317	2.11E-08
USP32	0.582889154	2.15E-08
INPP5A	0.582721076	2.18E-08
GRSF1	-0.582218934	2.25E-08
SLC22A1	0.581409612	2.38E-08
SNORD12B	0.581046562	2.44E-08
CDC123	0.580925404	2.46E-08
ARHGAP24	0.57953439	2.71E-08
CDK5R1	0.578766669	2.85E-08
ARHGEF6	-0.578623908	2.88E-08
CALM1	-0.578507068	2.90E-08
COA4	-0.578394613	2.92E-08
FOSL2	0.577668461	3.07E-08
ANTXR2	0.577666496	3.07E-08
PIGM	-0.577312417	3.15E-08
IL13RA1	0.577003573	3.21E-08
TRIB1	0.57611108	3.41E-08
ENTPD1	0.576094791	3.41E-08
ABHD5	0.575504088	3.55E-08
PHC2	0.57541193	3.57E-08
INHBB	0.574720057	3.74E-08
HNRNPM	-0.574434721	3.82E-08
VNN3P	0.573009269	4.20E-08
GAB1	0.571972331	4.50E-08
THOC5	0.571238888	4.72E-08
NXF1	-0.570888421	4.83E-08
C3orf86P	0.570736673	4.88E-08
LOC400499	0.570075494	5.10E-08
LINC02218	0.570003225	5.12E-08
HNRNPD	-0.56972502	5.21E-08
CAPN2	-0.56972502	5.21E-08

TMCC3	0.569392633	5.33E-08
SFPQ	-0.56914147	5.42E-08
ZNF487	0.568987974	5.47E-08
KCNJ2	0.568404413	5.69E-08
ETS2	0.568279821	5.73E-08
NLRP6	0.567676423	5.96E-08
LRRC8C	-0.567642075	5.98E-08
TIRAP	0.567613194	5.99E-08
MTFMT	0.567242884	6.13E-08
OTX1	0.564637894	7.26E-08
UBE2R2	0.564417411	7.37E-08
LINC02289	0.564410273	7.37E-08
NAP1L4	-0.564328891	7.41E-08
HNRNPA3	-0.564164496	7.49E-08
FOS	0.564030906	7.55E-08
DDX1	-0.564025393	7.56E-08
KIAA0040	0.563953084	7.59E-08
SRSF3	-0.563655102	7.74E-08
EWSR1	-0.56346898	7.83E-08
PSEN1	0.563342523	7.90E-08
PRDM8	0.562984882	8.08E-08
PELI2	0.56215936	8.52E-08
TNFRSF9	0.562032904	8.59E-08
RBM19	-0.561745607	8.75E-08
AQP9	0.561724022	8.76E-08
CNTNAP3	0.561527081	8.87E-08
RRAGC	0.561496244	8.89E-08
B9D2	0.561366237	8.96E-08
YPEL3-DT	0.560060193	9.74E-08
ABHD4	0.560034902	9.76E-08
PHF21A	0.559704088	9.97E-08
NUAK2	0.558967096	1.04E-07
MANSC1	0.558941804	1.05E-07
XPO6	0.55786952	1.12E-07
DUSP1	0.557706048	1.13E-07
SLC25A44	0.556902319	1.19E-07
CENPV	-0.556763216	1.20E-07
C3orf62	0.556712634	1.20E-07
NCL	-0.55651943	1.22E-07
CASP5	0.556472366	1.22E-07
IRF2BP2	-0.555760695	1.28E-07
HSPA9	-0.555533075	1.30E-07
AP5B1	0.55482843	1.36E-07
TSEN34	0.554673175	1.37E-07

TIAM2	0.554647884	1.37E-07
CD59	0.553896153	1.44E-07
PPP1R3B	0.553854714	1.44E-07
MINDY1	0.553450053	1.48E-07
POLR2C	0.553235077	1.50E-07
PDK3	0.552953376	1.52E-07
PPCDC	0.552805124	1.54E-07
KDM4B	0.552620264	1.56E-07
SLPI	0.551831408	1.63E-07
HSPA8	-0.551253193	1.69E-07
MGAM	0.551196935	1.70E-07
ELF2	0.550671489	1.76E-07
ARHGAP15	0.550649679	1.76E-07
EEF1A1	-0.550591935	1.76E-07
BICDL1	-0.550348386	1.79E-07
TARDBP	-0.5502232	1.80E-07
BEST1	0.550044892	1.82E-07
GNG5B	0.549807535	1.85E-07
KIF27	0.549741398	1.86E-07
KIAA0825	0.549589651	1.88E-07
CCDC13-AS2	0.54913441	1.93E-07
SPATA6	0.549049362	1.94E-07
GOSR2	-0.548982663	1.95E-07
IL1B	0.548669992	1.99E-07
AP2B1	-0.548568827	2.00E-07
MAP3K5	0.548534359	2.00E-07
TMEM121B	0.548173346	2.05E-07
NECAB2	0.547996307	2.07E-07
NSMCE1-DT	0.547977946	2.07E-07
TRIM44	-0.547718105	2.10E-07
ITPRIP	0.547367489	2.15E-07
BRCA1	0.546937537	2.21E-07
FCGR2A	0.546805749	2.23E-07
HNRNPA1	-0.546780058	2.23E-07
CAPRIN1	-0.546655876	2.25E-07
GTPBP4	-0.54652942	2.26E-07
MBOAT2	0.546507584	2.27E-07
LRP10	0.546504203	2.27E-07
MARK2	0.546461632	2.27E-07
KDM6B	0.546144313	2.32E-07
CTBS	0.546043147	2.33E-07
GMPS	-0.545992564	2.34E-07
CXCL1	0.545922432	2.35E-07
SIRPB1	0.54585117	2.36E-07

GART	-0.545694812	2.38E-07
METTL17	-0.545609742	2.39E-07
ZNF512	-0.545600546	2.39E-07
RALGAPA2	0.545268309	2.44E-07
LINC02631	0.545259111	2.44E-07
AAK1	-0.545138406	2.46E-07
ORM1	0.54501195	2.48E-07
YIPF1	0.544961368	2.49E-07
DENND10	0.544936077	2.49E-07
ERAP1	-0.544885494	2.50E-07
PDZD8	0.5441302	2.62E-07
GIMAP7	-0.544101468	2.62E-07
RBBP4	-0.54306453	2.79E-07
TNFSF14	0.542761036	2.84E-07
PADI2	0.542585075	2.87E-07
ARID3B	0.54240696	2.90E-07
LINC01270	0.541951719	2.99E-07
EIF5B	-0.541478034	3.07E-07
ZNF552	0.541243567	3.11E-07
TAF12	0.541120532	3.14E-07
LRRC58	-0.540687161	3.22E-07
ING3	0.540475603	3.26E-07
TLR1	0.540330688	3.29E-07
LSMEM1	0.540307793	3.29E-07
HSP90AB1	-0.540173124	3.32E-07
PRDM5	0.539422603	3.47E-07
MAEA	0.539397312	3.48E-07
TFEB	0.539220273	3.51E-07
ASPRV1	0.538739741	3.62E-07
ILF3	-0.538132753	3.75E-07
LENG8	-0.538060282	3.76E-07
CTBP2	0.537857951	3.81E-07
GMPR2	0.537829259	3.82E-07
CHIC2	0.537567101	3.88E-07
SET	-0.537449892	3.90E-07
ST20	0.537342877	3.93E-07
SLA	0.537248505	3.95E-07
RPL7L1	-0.536994651	4.01E-07
SPATA2	0.536893486	4.03E-07
BASP1	0.53682287	4.05E-07
TMPO	-0.536719842	4.08E-07
GPR27	0.536438245	4.14E-07
MAP3K8	0.536141531	4.22E-07
SCAT8	0.536134751	4.22E-07

THOC2	-0.535977137	4.26E-07
ACOX1	0.535920685	4.27E-07
GABARAPL1	0.535910519	4.27E-07
NSMCE3	-0.535780675	4.31E-07
RALB	0.535618085	4.35E-07
IRS2	0.535607023	4.35E-07
PRPF4B	-0.535581732	4.36E-07
UBE2G2	-0.535527763	4.37E-07
TMED10	-0.535458661	4.39E-07
NUP43	-0.535202362	4.46E-07
TRAF3IP3	-0.533565174	4.90E-07
CEACAM4	0.533419324	4.95E-07
CSRNP1	0.5328469	5.11E-07
CLEC4A	0.532723812	5.15E-07
PFKFB4	0.532192695	5.31E-07
DDX39B	-0.532142112	5.33E-07
SPN	-0.532138747	5.33E-07
GNL3	-0.532037583	5.36E-07
CNOT9	-0.531860545	5.42E-07
SH2B2	0.531734089	5.46E-07
CDC25B	-0.531455886	5.55E-07
FTH1P2	0.531386732	5.57E-07
TLR6	0.531187759	5.63E-07
SELENOH	-0.531032651	5.68E-07
NUCKS1	-0.530884259	5.73E-07
APTR	0.530848898	5.74E-07
CSF2RBP1	0.530508239	5.86E-07
PSENEN	0.530418948	5.89E-07
DOK4	0.530343075	5.91E-07
PPP1CC	-0.5302832	5.94E-07
MAP3K3	0.530043692	6.02E-07
KREMEN1	0.529913125	6.06E-07
TMEM216	0.529638271	6.16E-07
TMC8	-0.529508466	6.21E-07
SEC31B	-0.529398003	6.25E-07
ZFYVE1	0.529309484	6.28E-07
GIMAP6	-0.529094507	6.36E-07
RILPL1	0.528825605	6.46E-07
AK2	-0.528601327	6.54E-07
PLAU	0.528538098	6.56E-07
ZNF18	0.528525453	6.57E-07
TMEM43	0.528462944	6.59E-07
STARD7	-0.52806687	6.74E-07
TMEM185B	0.527788667	6.85E-07

HRH2	0.527694175	6.89E-07
KCNJ2-AS1	0.527611629	6.92E-07
ATP1A1	-0.527479198	6.97E-07
MTMR3	0.527406658	7.00E-07
CEP250	-0.527340095	7.03E-07
TUBA1A	0.527179033	7.10E-07
JAG1	0.526878185	7.22E-07
VPS37B	0.526827603	7.24E-07
LITAF	0.526803291	7.25E-07
MAP3K4	-0.526733099	7.28E-07
NFATC2IP	-0.526397653	7.42E-07
NECTIN1	0.526195324	7.51E-07
ARL11	0.526043577	7.57E-07
PLXDC2	0.525819281	7.67E-07
KDM5B	0.5256895	7.73E-07
TTLL4	0.5256895	7.73E-07
PCTP	0.5256895	7.73E-07
SNX5	-0.525573014	7.78E-07
CYSTM1	0.525541076	7.79E-07
IFITM2	0.525531664	7.80E-07
LINC02649	0.525266193	7.91E-07
EYS	0.524969334	8.05E-07
P3H3	-0.524878293	8.09E-07
TNFRSF25	-0.524349069	8.34E-07
LINC00654	0.524146739	8.43E-07
CNOT2	-0.524128076	8.44E-07
B3GNTL1	0.523868537	8.57E-07
SNORD89	0.523868537	8.57E-07
TBL1X	0.523783329	8.61E-07
CCT6A	-0.523723412	8.64E-07
CFAP58-DT	0.523615625	8.69E-07
TRA2B	-0.523438587	8.78E-07
TST	0.523236257	8.88E-07
TMEM11	0.523135093	8.93E-07
FUBP1	-0.522907472	9.05E-07
XKR8	0.522860196	9.07E-07
PLIN4	0.522860196	9.07E-07
TCERG1	-0.522705143	9.15E-07
ST3GAL4	0.522632574	9.19E-07
RAB5A	0.522603978	9.20E-07
FEZ2	0.522581991	9.21E-07
SLC9A8	0.522534713	9.24E-07
LTBP2	0.522379661	9.32E-07
MPHOSPH8	-0.522167986	9.43E-07

TOP2B	-0.52215534	9.44E-07
SLC22A4	0.522148737	9.44E-07
NONO	-0.522000291	9.52E-07
TNFRSF1A	0.521940363	9.55E-07
IFT56	0.521921117	9.56E-07
RMDN3	-0.52180126	9.63E-07
PLIN5	0.521238255	9.94E-07
PTP4A2	-0.521123825	1.00E-06
FAM169BP	0.521077158	1.00E-06
TLR8	0.520922645	1.01E-06
CACNG8	-0.520814892	1.02E-06
PTMA	-0.520800006	1.02E-06
BCORL1	0.520783014	1.02E-06
EGLN1	0.520638374	1.03E-06
DEDD	0.520369	1.04E-06
HNRNPDL	-0.520334353	1.05E-06
ARF6	-0.520034144	1.06E-06
BIRC3	-0.52002428	1.06E-06
CCT4	-0.519888465	1.07E-06
WDR20	0.519847241	1.07E-06
HSPA1L	0.519695494	1.08E-06
SPRY3	0.519493165	1.10E-06
HNRNPL	-0.519483804	1.10E-06
PCSK7	-0.519037924	1.12E-06
DHX9	-0.518990623	1.13E-06
SMIM3	0.518835595	1.14E-06
ARRDC4	0.518728344	1.14E-06
NATD1	0.518709139	1.14E-06
MED11	0.518633265	1.15E-06
HAPLN3	-0.518510088	1.16E-06
IPO5	-0.518380354	1.17E-06
TIAL1	-0.518320403	1.17E-06
TLK2	0.518203316	1.18E-06
FAM174A	0.516979963	1.26E-06
EIF3J	-0.516483516	1.30E-06
SDCBPP3	0.516423554	1.30E-06
LRRK1	0.516132704	1.32E-06
LATS2	0.51609803	1.32E-06
BIN3	0.516078858	1.32E-06
OSER1	0.516028276	1.33E-06
HCAR2	0.515876529	1.34E-06
ORAI3	0.515829208	1.34E-06
PTGES3	-0.515785148	1.35E-06
PLEKHM3	0.515598326	1.36E-06

PLXNC1	0.51538452	1.38E-06
CCT2	-0.515168376	1.39E-06
RRAGD	0.515117794	1.40E-06
RANBP1	-0.515061079	1.40E-06
PIRAT1	0.515016629	1.40E-06
RPL15	-0.514716389	1.43E-06
ATF6	0.514662553	1.43E-06
RNF144B	0.51461197	1.44E-06
DDX21	-0.514412894	1.45E-06
SIPA1L2	0.514147335	1.47E-06
HNRNPH1	-0.514144442	1.47E-06
RAN	-0.513853235	1.50E-06
CANX	-0.51384454	1.50E-06
DHRS13	0.513502406	1.53E-06
DNMBP	0.513350658	1.54E-06
HIVEP1	0.513246247	1.55E-06
BAZ1B	-0.51319891	1.55E-06
SUGP2	-0.513075697	1.56E-06
ATP5F1A	-0.512766038	1.59E-06
LIMD1	-0.512689842	1.60E-06
UBXN2B	0.512655146	1.60E-06
CANT1	0.512604564	1.60E-06
SLC16A5	0.512591918	1.60E-06
F2RL1	0.512538095	1.61E-06
SLC49A4	0.51252869	1.61E-06
EFHC2	0.512512804	1.61E-06
EVL	-0.51243693	1.62E-06
RBM17	-0.512424599	1.62E-06
SKIC3	-0.512411639	1.62E-06
OSGIN2	0.512386348	1.62E-06
PLXNA2	0.512361057	1.62E-06
GSK3B	0.512203141	1.64E-06
SF1	-0.512121404	1.65E-06
E2F3	0.512101975	1.65E-06
SEMA4B	0.512022863	1.65E-06
ERGIC1	0.511804651	1.67E-06
SRSF11	-0.511754069	1.68E-06
ZNF586	0.511697312	1.68E-06
TMEM88	0.51149498	1.70E-06
CAND1	-0.511450575	1.71E-06
ALDH1A2	0.511425284	1.71E-06
MAPKAPK5-AS1	-0.511368522	1.71E-06
PRUNE1	0.511321172	1.72E-06
TGFA	0.511289415	1.72E-06

ZNF207	-0.511264124	1.72E-06
JPT1	0.511200895	1.73E-06
MLEC	-0.511175604	1.73E-06
KCTD2	0.510973273	1.75E-06
KIAA0319	0.510944751	1.75E-06
LGALSL	0.510717131	1.78E-06
PAICS	-0.510062786	1.84E-06
ROPN1L	0.509933105	1.85E-06
AGK	-0.509911038	1.86E-06
SLC22A14	0.5098731	1.86E-06
C5AR1	0.509824266	1.86E-06
S100A11	0.509811618	1.87E-06
PELATON	0.509705484	1.88E-06
KIAA0232	0.509629611	1.88E-06
CREBL2	-0.509326117	1.92E-06
RNF111	0.5092914	1.92E-06
MAPRE3	0.509224952	1.93E-06
VDR	0.508972041	1.95E-06
TESK2	0.508693838	1.98E-06
EIF3A	-0.50831447	2.02E-06
TIMP2	0.50816293	2.04E-06
METAP2	-0.508067984	2.05E-06
IKBKB	-0.507913023	2.07E-06
PPM1H	0.507884521	2.07E-06
TXNRD1	0.507859229	2.07E-06
LRPPRC	-0.507758065	2.08E-06
ZC3H8	-0.507758065	2.08E-06
ZC3H7B	-0.507479862	2.12E-06
LINC00921	0.507445134	2.12E-06
UBP1	-0.507353406	2.13E-06
APOL3	-0.507201659	2.15E-06
MAGEH1	-0.507144844	2.15E-06
TBC1D14	0.507049912	2.17E-06
BASP1-AS1	0.507015181	2.17E-06
ZNF746	0.506977244	2.17E-06
ZNF185	0.506948747	2.18E-06
SRSF2	-0.506822292	2.19E-06
PPP1R15A	0.506822292	2.19E-06
RCC2	-0.506771709	2.20E-06
SUPT16H	-0.506686394	2.21E-06
MIR3945HG	0.506559937	2.22E-06
EIF4B	-0.506525203	2.23E-06
COP1	0.506417633	2.24E-06
LRG1	0.506291177	2.26E-06

SRSF7	-0.506190013	2.27E-06
NEK6	0.506012974	2.29E-06
SBF2	0.50596879	2.29E-06
API5	-0.505937101	2.30E-06
JADE1	0.505915009	2.30E-06
RBM47	0.50591181	2.30E-06
LINC00862	0.505810645	2.31E-06
GLUD1	-0.505715876	2.33E-06
ELL	0.50570948	2.33E-06
UQCRHL	0.505380695	2.37E-06
N4BP2L1	-0.505304822	2.38E-06
TRIM9	0.50528592	2.38E-06
MTA2	-0.505232143	2.39E-06
NNT	-0.505203657	2.39E-06
FRAT1	0.505178366	2.39E-06
ZBTB34	0.505045651	2.41E-06
IPO7	-0.505001328	2.42E-06
HNRNPR	-0.504966584	2.42E-06
KCTD21	0.504900163	2.43E-06
STX3	0.504716862	2.45E-06
PURA	-0.504640987	2.46E-06
ECHDC3	0.504192011	2.52E-06
PDHA1	-0.503992868	2.55E-06
PDS5A	-0.503904348	2.56E-06
FAM200B	0.503727309	2.59E-06
MARCKS	0.503717848	2.59E-06
RPL3	-0.503607221	2.60E-06
TMLHE	0.503509149	2.62E-06
B4GALT5	0.503196191	2.66E-06
SSRP1	-0.502927452	2.70E-06
FKBP9	0.502851579	2.71E-06
G3BP1	-0.502630314	2.74E-06
CHD3	-0.502159247	2.81E-06
IL1R1	0.502070727	2.82E-06
WDFY3-AS2	0.501956916	2.84E-06
CNTRL	-0.501890515	2.85E-06
DHX34	0.501779877	2.87E-06
PPP1R3D	0.501409982	2.92E-06
CNEP1R1	0.501232944	2.95E-06
SRSF6	-0.501182362	2.96E-06
SEC31A	-0.501040119	2.98E-06
ARL4C	-0.500882035	3.01E-06
MSRB3	0.500828286	3.01E-06
NPM1	-0.500777703	3.02E-06

DOCK10	-0.50060383	3.05E-06
QPCT	0.500569058	3.06E-06
RPL13A	-0.500534286	3.06E-06
SRP72	-0.500464728	3.07E-06
SAMHD1	-0.50040785	3.08E-06
LAT	-0.500379372	3.09E-06
JDP2	0.500366727	3.09E-06
SNHG3	-0.500347754	3.09E-06
DNAJC3	0.500316144	3.10E-06
ZNF467	0.500221298	3.11E-06
TRIM14	-0.499993677	3.15E-06
SUSD6	0.499652238	3.21E-06
RBMX	-0.499633274	3.21E-06
TUFT1	0.499630114	3.21E-06
DYNC1H1	-0.499389847	3.25E-06
C1RL	0.499386689	3.25E-06
CDA	0.499361398	3.26E-06
LPXN	-0.499326618	3.26E-06
EXOSC1	-0.499260233	3.27E-06
MCUB	-0.499209651	3.28E-06
ENGASE	-0.499098997	3.30E-06
KIF3C	0.499098997	3.30E-06
SNRNP70	-0.499086351	3.30E-06
FCAR	0.498855575	3.34E-06
ST6GALNAC2	0.49875441	3.36E-06
KPNB1-DT	0.498653245	3.38E-06
GOLGA7B	-0.498567879	3.39E-06
TMEM234	0.498349752	3.43E-06
LUC7L3	-0.498330762	3.44E-06
TMEM138	-0.498172713	3.46E-06
PITPNA	0.498014619	3.49E-06
AKIRIN2	0.497926098	3.51E-06
ACSS3	0.497869219	3.52E-06
HBP1	0.497647892	3.56E-06
HSDL2	0.497641599	3.56E-06
MAP1LC3B	0.497559372	3.58E-06
CASP9	0.49754358	3.58E-06
SP140L	-0.497489852	3.59E-06
SEPTIN2	-0.497477143	3.59E-06
TMEM245	-0.497413978	3.60E-06
RABL2B	-0.497085193	3.67E-06
PBLD	0.497028251	3.68E-06
DIS3L2	-0.496999817	3.68E-06
MAT2A	-0.496933446	3.70E-06

CCT8	-0.496661524	3.75E-06
SHROOM4	0.496645738	3.75E-06
COQ10B	0.496607801	3.76E-06
SLC22A15	0.496503497	3.78E-06
NLRX1	0.49649399	3.78E-06
RBM26	-0.496478205	3.78E-06
PHACTR4	-0.496452914	3.79E-06
DHRSX	0.496326458	3.81E-06
CRK	0.496200003	3.84E-06
LINC00861	-0.49611462	3.86E-06
PANX2	0.495972382	3.88E-06
KCMF1	0.495684668	3.94E-06
MTND6P11	0.495423788	4.00E-06
NFE4	0.495390685	4.00E-06
IL1RAP	0.495340103	4.01E-06
MSL1	0.495111868	4.06E-06
ZNF143	0.494887991	4.11E-06
ACTN1	0.494815115	4.12E-06
PHF24	0.494568312	4.18E-06
TADA2B	0.494420102	4.21E-06
COG2	-0.494293645	4.24E-06
STK35	0.494258833	4.24E-06
LNCATV	0.494243062	4.25E-06
GPX3	0.494217771	4.25E-06
RTN2	0.494176709	4.26E-06
PGLYRP1	0.494116606	4.28E-06
AATK	0.494116606	4.28E-06
BMS1	-0.494081793	4.28E-06
TOMM70	-0.494059625	4.29E-06
TRIM56	-0.493888984	4.33E-06
ZNF230	0.493847924	4.33E-06
FUS	-0.493803586	4.34E-06
ANAPC7	-0.493765649	4.35E-06
TCF7	-0.493699299	4.37E-06
C1orf226	0.493690697	4.37E-06
TNRC6A	-0.493572842	4.40E-06
MLLT6	-0.493398923	4.44E-06
RCOR1	0.493395803	4.44E-06
TLE5	-0.493342101	4.45E-06
SEPTIN6	-0.493297757	4.46E-06
ALOX15	-0.493174073	4.49E-06
PLEKHG4	-0.493168181	4.49E-06
PKNOX1	0.493089189	4.51E-06
RFC2	0.49288686	4.55E-06

CCAR1	-0.492861569	4.56E-06
ETS1	-0.492848743	4.56E-06
LARP1	-0.492776165	4.58E-06
DCAF4L1	0.492649709	4.61E-06
APH1B	0.492406328	4.67E-06
DNMT1	-0.492178707	4.72E-06
SLC15A3	0.49215964	4.73E-06
RARA-AS1	0.491875213	4.80E-06
DYNC1LI1	0.491647593	4.85E-06
SDCBP	0.491539936	4.88E-06
PPIA	-0.491486308	4.89E-06
NUDT16L2P	0.491470555	4.90E-06
HMG20A	-0.49142308	4.91E-06
PARP1	-0.491397788	4.92E-06
PEAK3	0.491318808	4.94E-06
DENND2D	-0.491233395	4.96E-06
NCBP2	-0.491040605	5.01E-06
PUM3	-0.491024628	5.01E-06
HAPSTR1	0.490869774	5.05E-06
CLP1	0.490759066	5.08E-06
TTC17	-0.49070538	5.09E-06
UBE2B	0.490642151	5.11E-06
ASPH	0.490560073	5.13E-06
KTN1	-0.490455566	5.16E-06
LOC122526782	0.490408326	5.17E-06
BCS1L	-0.49011103	5.25E-06
TESMIN	0.489978376	5.28E-06
NFKBIA	0.489956183	5.29E-06
IL23A	-0.489459641	5.43E-06
HSPD1	-0.48942197	5.44E-06
SYNCRIP	-0.489346097	5.46E-06
ZNF200	0.489320806	5.46E-06
TRAF1	-0.489169059	5.51E-06
ATP8B2	-0.488691617	5.64E-06
BTBD10	0.488688527	5.64E-06
SGPL1	0.488612653	5.66E-06
SH2B1	-0.48844488	5.71E-06
HPN	0.488429582	5.72E-06
ARHGAP26-IT1	0.488397385	5.73E-06
RAB31	0.488340626	5.74E-06
FAM174B	0.488334155	5.74E-06
CBX4	0.488030956	5.83E-06
TTC27	-0.487803336	5.90E-06
NOP58	-0.487730546	5.92E-06

ZBTB40	-0.487654672	5.94E-06
TPST1	0.487474551	6.00E-06
GABBR1	-0.487474551	6.00E-06
DNAAF11	0.487221639	6.08E-06
PTCD3	-0.487171057	6.09E-06
FRMD4B	0.486994019	6.15E-06
GTF3A	-0.486782122	6.21E-06
SYTL3	0.486696679	6.24E-06
RABIF	0.486693602	6.24E-06
ADCY7	-0.486617728	6.26E-06
TMEM199	0.486538778	6.29E-06
CDC26	0.486225712	6.39E-06
ACP3	0.486108828	6.43E-06
ALPL	0.486089684	6.43E-06
MCU	0.486032954	6.45E-06
RBM6	-0.485821051	6.52E-06
CDKN2D	0.485808406	6.52E-06
MTREX	-0.485628296	6.58E-06
NINJ2	0.485451258	6.64E-06
EIF2D	-0.485340516	6.68E-06
DHX40	0.485201414	6.73E-06
NEK9	-0.48503702	6.78E-06
ARHGAP19	0.484999083	6.79E-06
ANAPC5	-0.484761881	6.87E-06
ABCE1	-0.484743105	6.88E-06
NOL8	-0.484667232	6.91E-06
EFCAB14	-0.484556485	6.95E-06
TARBP1	-0.484531194	6.95E-06
LOC105373780	0.484490193	6.97E-06
TPD52L2	0.484303175	7.03E-06
INPP5E	-0.484287864	7.04E-06
UBE2D1	0.484287864	7.04E-06
SDHAF2	0.48427828	7.04E-06
DGKA	-0.484202406	7.07E-06
MBTPS1	-0.484034952	7.13E-06
GATAD1	-0.483911556	7.17E-06
ITM2A	-0.483857914	7.19E-06
RNU6-226P	0.483782041	7.22E-06
TFAP4	-0.483706167	7.25E-06
ECHDC2	-0.483630294	7.27E-06
KDM4C	-0.483557478	7.30E-06
KLF5	0.48340573	7.36E-06
SIAH1	0.483320266	7.39E-06
STX11	0.483225635	7.42E-06

SRRT	-0.483143226	7.45E-06
FLNA	-0.483142374	7.45E-06
BUD31	0.483048597	7.49E-06
ABITRAM	0.483023306	7.50E-06
CCND2	-0.482998015	7.51E-06
RNASEL	0.482798738	7.58E-06
FYN	-0.482770394	7.59E-06
ESYT1	-0.482745103	7.60E-06
FUT7	0.48266923	7.63E-06
NAP1L1	-0.482564573	7.67E-06
TPK1	0.482542774	7.68E-06
SNORC	0.482409328	7.73E-06
DUSP18	0.482340444	7.76E-06
STT3B	-0.482308607	7.77E-06
RPUSD4	-0.482220087	7.80E-06
NUMA1	-0.482087533	7.85E-06
TPP2	-0.48203695	7.87E-06
ZNF319	0.482014707	7.88E-06
CLEC4E	0.481657583	8.02E-06
MYBPC3	0.481581709	8.05E-06
NSFL1C	0.481395069	8.13E-06
CHSY1	0.481382424	8.13E-06
PLPP3	0.481354089	8.15E-06
RNF125	-0.481303507	8.17E-06
H3-3A	0.481129511	8.24E-06
NMNAT1	0.480901889	8.33E-06
LAMA5	-0.48085193	8.35E-06
ESYT2	-0.480686912	8.42E-06
SPON1	-0.480679369	8.42E-06
PIP4P2	0.480645936	8.44E-06
LOC102723701	0.480615758	8.45E-06
FBXO34	0.480544772	8.48E-06
SFRP1	0.480537287	8.48E-06
RAPGEF2	0.480443607	8.52E-06
IQSEC1	0.480443113	8.52E-06
NPR2	-0.480390054	8.54E-06
KAZN	0.480317151	8.57E-06
GGA2	-0.480266569	8.60E-06
NOL6	-0.480231669	8.61E-06
KATNIP	0.480165404	8.64E-06
CEBPD	0.480140113	8.65E-06
TSEN15	-0.480089531	8.67E-06
SF3B3	-0.48006424	8.68E-06
ZNF706	-0.479956499	8.73E-06

IL7R	-0.479822954	8.79E-06
IL11RA	-0.479583707	8.89E-06
LOC124909446	0.47949822	8.93E-06
PLCG1	-0.479485574	8.93E-06
GBE1	0.479457252	8.94E-06
PDIA3	-0.479456213	8.95E-06
FPR2	0.479444086	8.95E-06
LARS1	-0.479280213	9.02E-06
LINC00877	0.479270598	9.03E-06
MSRB1	0.479096588	9.10E-06
ZNF516	0.479030331	9.13E-06
RIC3	-0.478900846	9.19E-06
XPOT	-0.478853291	9.21E-06
GPR141	0.478850264	9.22E-06
TLE3	0.478837083	9.22E-06
LMO4	0.478834055	9.22E-06
LRFN1	0.478824972	9.23E-06
IKBIP	0.478824972	9.23E-06
CCDC88C	-0.47877439	9.25E-06
DDIT3	0.478723808	9.27E-06
SLC7A6	-0.478698517	9.28E-06
KDM4A	0.478657014	9.30E-06
OLAH	0.478653987	9.31E-06
ATF7IP2	-0.478597352	9.33E-06
IFNAR1	0.478590759	9.33E-06
TFIP11-DT	-0.478554147	9.35E-06
LXN	0.478420314	9.41E-06
LIMK2	0.478420314	9.41E-06
MORC2	-0.478350489	9.44E-06
DDX27	-0.478284237	9.48E-06
KLHDC4	-0.478132489	9.55E-06
UQCC1	-0.47811022	9.56E-06
BRD7	-0.478081906	9.57E-06
RPLP0	-0.477945268	9.63E-06
SMPD3	-0.47786693	9.67E-06
CAMKK1	0.477813326	9.70E-06
FAM131A	0.477762744	9.72E-06
TRIQK	0.477762744	9.72E-06
CINP	0.47745925	9.87E-06
SNX11	0.477433958	9.88E-06
DHX30	-0.477411686	9.89E-06
BNIP3	-0.477070253	1.01E-05
EML4	-0.4770293	1.01E-05
ZFAND3	0.477004009	1.01E-05

OIP5-AS1	-0.476969088	1.01E-05
RABL2A	-0.476928135	1.01E-05
BTG2-DT	0.476917338	1.01E-05
IDH3A	-0.476852262	1.02E-05
KLHL18	0.476779403	1.02E-05
SRPK1	0.476769772	1.02E-05
MIDN	0.476548768	1.03E-05
SF3A3	-0.476523477	1.03E-05
ANKRD52	-0.476472894	1.04E-05
TMEM50B	-0.476270565	1.05E-05
ZNF674	0.476068236	1.06E-05
ATOSB	0.47594178	1.06E-05
TNFAIP8L2	0.475916489	1.06E-05
RAB3D	0.475894206	1.06E-05
RPS23	-0.475815324	1.07E-05
ZNF687	0.475805687	1.07E-05
CRYGS	-0.475729813	1.07E-05
FPR1	0.475679724	1.08E-05
DNAJC3-DT	0.47567923	1.08E-05
MGAM2	0.47551183	1.08E-05
FAM98B	-0.475186049	1.10E-05
MRPS5	-0.474983719	1.11E-05
KBTBD7	0.474930133	1.12E-05
PPP1R16B	-0.474923493	1.12E-05
ATXN7L3B	-0.474800038	1.12E-05
TMEM147-AS1	-0.474689227	1.13E-05
HACD3	-0.474677222	1.13E-05
TMPPE	0.474677222	1.13E-05
PHTF1	0.474323145	1.15E-05
PPP3R1	0.474294205	1.15E-05
DARS1	-0.474288207	1.15E-05
RNF146	0.474095525	1.16E-05
RAI2	0.474041289	1.17E-05
PDCD11	-0.47399436	1.17E-05
SOS2	0.473820318	1.18E-05
CFLAR-AS1	0.473655924	1.19E-05
EZR	-0.47360168	1.19E-05
UBTF	-0.473539119	1.19E-05
RN7SL271P	0.473511494	1.20E-05
FASTKD2	-0.473469233	1.20E-05
EIF4A2	-0.473406004	1.20E-05
POLR3E	-0.473317484	1.21E-05
HTRA1	0.473165735	1.22E-05
RSL1D1	-0.473159752	1.22E-05

SULT1B1	0.473086869	1.22E-05
NAE1	-0.472957422	1.23E-05
IP6K1	0.472922476	1.23E-05
TRAM1	-0.47290983	1.23E-05
PRR13	0.472862237	1.23E-05
DHRS7B	0.472808665	1.24E-05
TRAF6	0.472732791	1.24E-05
KRI1	-0.472552764	1.25E-05
MAN2A2	0.472502181	1.25E-05
MAPK3	0.472444928	1.26E-05
LOC124906608	0.472201673	1.27E-05
NDUFA2	0.472122814	1.28E-05
UBFD1	-0.472049925	1.28E-05
RPSA	-0.472021649	1.28E-05
FADD	0.471971067	1.29E-05
NAA15	-0.471920485	1.29E-05
ACIN1	-0.471887804	1.29E-05
TTC39C	-0.471844611	1.30E-05
NSUN7	0.471844611	1.30E-05
FAM111A	-0.471736766	1.30E-05
IGF1R	0.471718155	1.30E-05
HMGN1	-0.471667573	1.31E-05
XPO1	-0.471616991	1.31E-05
NFYC	0.471496497	1.32E-05
MLLT3	-0.471471206	1.32E-05
SNORD19C	-0.471462754	1.32E-05
RNF149	0.471451465	1.32E-05
B3GNT5	0.471414661	1.32E-05
CBLL1-AS1	0.471111167	1.34E-05
BRD9	-0.470930416	1.35E-05
MTAP	-0.470892479	1.36E-05
ST6GALNAC3	0.470757091	1.36E-05
TMSB4X	-0.470734925	1.37E-05
MRS2	-0.470478888	1.38E-05
NCBP1	-0.470408963	1.39E-05
CPNE2	0.470403015	1.39E-05
SMIM27	0.470317469	1.39E-05
BMX	0.47030185	1.39E-05
NR6A1	0.47030185	1.39E-05
RAD17	-0.470279532	1.40E-05
CHMP7	-0.470105466	1.41E-05
ZNF438	0.47005191	1.41E-05
RELL1	0.469897191	1.42E-05
IPO9	-0.469751385	1.43E-05

EXT1	0.46964428	1.44E-05
PIEZO1	-0.469369045	1.46E-05
RIT1	0.469192005	1.47E-05
SLCO3A1	0.469128777	1.47E-05
TRMT1	-0.469062583	1.48E-05
ARID5A	0.469012001	1.48E-05
TBC1D30	0.468989675	1.48E-05
RPL4	-0.468865396	1.49E-05
PHF10	-0.468825281	1.50E-05
UTP15	-0.46878438	1.50E-05
BANP	0.468733798	1.50E-05
SSH2	0.468684596	1.51E-05
CDK5RAP1	-0.468632633	1.51E-05
ALPK3	0.468445911	1.52E-05
LUC7L	-0.468284479	1.54E-05
F11R	0.468218289	1.54E-05
ANO10	0.468202683	1.54E-05
NUP210	-0.468050936	1.55E-05
MTO1	-0.467924481	1.56E-05
TSPOAP1-AS1	-0.467899189	1.56E-05
CLEC1A	0.467798025	1.57E-05
LIN7A	0.4677504	1.57E-05
RAB5C	0.467623943	1.58E-05
PTENP1	0.467620987	1.58E-05
TBCD	-0.467598652	1.59E-05
TUBGCP4	-0.467570404	1.59E-05
AHNAK	-0.467536739	1.59E-05
SRRM2	-0.467435568	1.60E-05
GDE1	0.46726691	1.61E-05
SPTAN1	-0.467193991	1.62E-05
MRPL9	-0.467184299	1.62E-05
ATXN1	0.467181345	1.62E-05
CCDC28A	0.467140454	1.62E-05
LINC02035	0.467042243	1.63E-05
ERF	0.466963416	1.63E-05
CARMIL2	-0.466938125	1.64E-05
NAT10	-0.466912834	1.64E-05
MCM3AP	-0.466890495	1.64E-05
ADM	0.466836961	1.64E-05
DTYMK	-0.466786378	1.65E-05
NKTR	-0.46676699	1.65E-05
CD84	-0.466685214	1.66E-05
RTL8B	0.466596091	1.66E-05
METTL25	0.466508175	1.67E-05

SNHG1	-0.466482884	1.67E-05
ILF2	-0.466384668	1.68E-05
TC2N	-0.466305846	1.69E-05
HLA-DRA	-0.466298245	1.69E-05
SLFN13	-0.46617939	1.70E-05
CSE1L	-0.466052934	1.71E-05
SIGLEC8	-0.465959047	1.71E-05
MTCO2P33	0.465901187	1.72E-05
ZNF37A	-0.465770921	1.73E-05
CCDC17	0.465448889	1.76E-05
GCM1	0.465445946	1.76E-05
ZRANB2	-0.465420655	1.76E-05
MALT1	-0.465398307	1.76E-05
SLC45A4	0.465335078	1.77E-05
IMP4	-0.465243617	1.77E-05
SEPTIN9	-0.465170685	1.78E-05
SSB	-0.465148335	1.78E-05
CDC16	-0.465145394	1.78E-05
MRPS21	-0.465056874	1.79E-05
CNPY2	-0.465021877	1.79E-05
MYH3	-0.464968354	1.80E-05
ANKS1A	0.464813667	1.81E-05
NUDT16-DT	0.464813667	1.81E-05
C11orf54	0.464791315	1.81E-05
CCDC71L	0.464791315	1.81E-05
ARL3	0.464763085	1.81E-05
RAB43	0.464750705	1.81E-05
BICRAL	0.464740732	1.82E-05
POLR3D	-0.464737794	1.82E-05
SOCS3-DT	0.464736011	1.82E-05
TMCC1	0.46469015	1.82E-05
TXNL4B	0.464611338	1.83E-05
MTX1	0.464611338	1.83E-05
C1QBP	-0.464586047	1.83E-05
CDK19	0.464579276	1.83E-05
SLFN5	-0.464563693	1.83E-05
RN7SL491P	0.464545421	1.83E-05
CDH24	-0.464379879	1.85E-05
COX11	-0.464358426	1.85E-05
RNF145	0.464313716	1.85E-05
MPHOSPH10	-0.464171678	1.86E-05
DMAP1	-0.463953768	1.88E-05
AARSD1	-0.463918764	1.89E-05
DGCR11	0.463893473	1.89E-05

UBE3A	-0.46377673	1.90E-05
CA4	0.46377673	1.90E-05
SLC51A	0.463715516	1.90E-05
OSGEPL1	-0.463691142	1.91E-05
DDX24	-0.463678497	1.91E-05
BLMH	-0.463678497	1.91E-05
UBA2	-0.463650274	1.91E-05
RMI1	0.463624983	1.91E-05
CUL1	-0.463599691	1.92E-05
SIRPD	0.463599691	1.92E-05
SERTAD3	0.4635744	1.92E-05
RBPJ	0.463514103	1.92E-05
CFAP97	-0.463422653	1.93E-05
BTBD9	0.463362355	1.94E-05
TASL	0.463223253	1.95E-05
OLFM2	-0.463220324	1.95E-05
OPRL1	0.463119159	1.96E-05
DNAJC21	-0.463068577	1.96E-05
HARS1	-0.463033568	1.97E-05
SMAP2	0.463022106	1.97E-05
FAM98A	-0.462992703	1.97E-05
RASGRP3-AS1	-0.462992703	1.97E-05
CD96	-0.462891539	1.98E-05
CCNL2	-0.462849736	1.98E-05
RPS3	-0.462824444	1.99E-05
HSP90AA1	-0.462789433	1.99E-05
TARM1	0.462726891	1.99E-05
HNRNPH2	0.462714501	2.00E-05
ADH5	-0.462707706	2.00E-05
ABLIM1	-0.462704781	2.00E-05
NLRC5	-0.462692135	2.00E-05
TNNI2	0.46268921	2.00E-05
GFOD3P	-0.462682415	2.00E-05
MRPL35	-0.462593895	2.01E-05
PABPC1	-0.462509781	2.02E-05
PAPSS2	0.46248688	2.02E-05
TNFAIP1	0.462477159	2.02E-05
LANCL1	-0.462436298	2.02E-05
MACIR	0.462385716	2.03E-05
BCL3	0.462284551	2.04E-05
EIF3M	-0.462198954	2.04E-05
HSD17B13	0.462183386	2.05E-05
LINC00173	0.462173663	2.05E-05
MAP1LC3B2	0.462148372	2.05E-05

WDR47	0.462082222	2.06E-05
CSNK2A1	-0.462082222	2.06E-05
DDB1	-0.461971332	2.07E-05
HTATIP2	0.461652272	2.10E-05
HAUS4	0.461525816	2.11E-05
BCL11B	-0.461525816	2.11E-05
RCAN3	-0.461490797	2.11E-05
INMT	-0.461474141	2.11E-05
LOC124904411	0.461414923	2.12E-05
LINC01191	0.461359449	2.13E-05
HNRNPU	-0.46130499	2.13E-05
RASGRP4	0.461304029	2.13E-05
NEMF	-0.461288467	2.13E-05
MYLIP	0.461247613	2.14E-05
HYCC2	0.461187301	2.14E-05
SRP68	-0.46117174	2.14E-05
ADGRG3	0.461164926	2.15E-05
VPS13C	-0.461095866	2.15E-05
NR4A2	0.461045284	2.16E-05
ZNF37BP	-0.461045284	2.16E-05
WDR75	-0.461019993	2.16E-05
PRKCZ	0.46096941	2.16E-05
PDCD2	-0.460757349	2.19E-05
ABAT	0.460732057	2.19E-05
ZBTB4	-0.460706766	2.19E-05
RPL10A	-0.460665916	2.20E-05
RRP1B	-0.460656183	2.20E-05
LOC100421569	0.46046941	2.22E-05
ADCY4	0.460438296	2.22E-05
ZAP70	-0.460413005	2.22E-05
IDO1	-0.460327396	2.23E-05
SRSF5	-0.460289459	2.23E-05
FASTKD5	0.460210675	2.24E-05
ARG1	0.460185384	2.25E-05
ARFGAP3	0.460008346	2.26E-05
CARD11	-0.459957764	2.27E-05
NDUFB6	0.45988189	2.28E-05
PPP3CA	0.459799186	2.29E-05
NCKAP1L	-0.459659062	2.30E-05
SLC2A3	0.4593964	2.33E-05
APMAP	0.459376067	2.33E-05
C15orf61	-0.459040441	2.37E-05
ZCWPW1	0.458920826	2.38E-05
STAT2	-0.458913984	2.38E-05

IL6R	0.458770712	2.40E-05
PLEKHO2	0.458714552	2.40E-05
NOP10	0.458642623	2.41E-05
PRMT5	0.458617332	2.41E-05
RPL19	-0.45851407	2.43E-05
RPL8	-0.458202925	2.46E-05
TXLNA	-0.458086218	2.47E-05
ZNF75A	-0.458086218	2.47E-05
POLD3	0.458079365	2.47E-05
BCCIP	-0.458035635	2.48E-05
SSH1	0.458025886	2.48E-05
MARCHF5	0.457959762	2.49E-05
RUBCNL	0.457934471	2.49E-05
TBC1D4	-0.457782724	2.51E-05
IL18	0.457725284	2.52E-05
SUSD4	-0.457681559	2.52E-05
SMAD3	-0.457646516	2.52E-05
CD2AP	-0.457624118	2.53E-05
RASA3	-0.457545351	2.54E-05
EVI5	0.45743154	2.55E-05
RPL6	-0.457428647	2.55E-05
MLX	0.457380957	2.56E-05
PGM1	0.457251609	2.57E-05
HEATR1	-0.457165981	2.58E-05
MED18	0.457150444	2.58E-05
TP53I13	-0.457150444	2.58E-05
RACK1	-0.456903311	2.61E-05
TRPM6	0.456849839	2.62E-05
TCP11L2	0.456773965	2.63E-05
ARID3A	0.456751562	2.63E-05
MAP9	-0.456745786	2.63E-05
DNM1L	-0.456720495	2.63E-05
SLC11A1	0.4567194	2.63E-05
PHF14	-0.456695204	2.64E-05
EAPP	0.456442292	2.67E-05
ELK4	-0.456417001	2.67E-05
SIGLEC9	0.456417001	2.67E-05
SS18	-0.456321607	2.68E-05
PXK	0.456315836	2.68E-05
MFSD13A	0.456268139	2.69E-05
SLIT1	0.456164089	2.70E-05
EIF3H	-0.456154328	2.70E-05
URI1	-0.456037633	2.72E-05
RABEPK	0.455980172	2.72E-05

DLG1	-0.45596176	2.73E-05
UBE2C	0.455916943	2.73E-05
POLR2J	0.455885886	2.74E-05
PCED1B-AS1	-0.455860595	2.74E-05
LPCAT3	0.45575943	2.75E-05
FAHD2A	-0.455661147	2.76E-05
TESPA1	-0.455557101	2.78E-05
ARMH2	0.455540451	2.78E-05
IRF8	-0.45553181	2.78E-05
SLC19A1	0.455380063	2.80E-05
VAMP3	0.455370297	2.80E-05
LILRA2	0.45536053	2.80E-05
MRPS25	-0.455345005	2.80E-05
PRKACB	-0.455329481	2.81E-05
RPL27A	-0.455325472	2.81E-05
ZNF146	-0.455278898	2.81E-05
PTGS2	0.455228316	2.82E-05
RILPL2	0.455228316	2.82E-05
DUSP11	0.45510186	2.84E-05
ZNF486	0.45510186	2.84E-05
PLAGL1	0.455051278	2.84E-05
CD28	-0.455025987	2.85E-05
EIF2B5	-0.454940344	2.86E-05
LDLRAP1	-0.454798366	2.88E-05
BAZ1A-AS1	0.454798366	2.88E-05
CNTNAP3B	0.454681501	2.89E-05
LTB4R	0.454674785	2.89E-05
HTT	-0.454667889	2.89E-05
UBTD1	0.454596037	2.90E-05
ZNF396	0.454494872	2.92E-05
TARS3	-0.454462682	2.92E-05
ATXN2L	-0.454421872	2.93E-05
C11orf21	-0.454393708	2.93E-05
FNBP4	-0.454343125	2.94E-05
RAMAC	0.454343125	2.94E-05
GEMIN8	-0.454317834	2.94E-05
RNU7-45P	0.454317834	2.94E-05
CCDC152	-0.454270124	2.95E-05
HEBP2	0.454216669	2.95E-05
MRPS30	-0.454080439	2.97E-05
HSPA6	0.453979274	2.98E-05
MPC2	0.453928691	2.99E-05
FXYD7	-0.453888749	3.00E-05
COQ7	0.453776943	3.01E-05

N6AMT1	-0.453761428	3.01E-05
WDR33	-0.453726361	3.02E-05
PRMT7	-0.453675778	3.03E-05
SPACA6	0.453660264	3.03E-05
EXOSC8	-0.453650487	3.03E-05
RBMXL1	0.453609681	3.04E-05
AK1	-0.453590126	3.04E-05
MPZL3	0.453514252	3.05E-05
TRAF5	-0.453483226	3.05E-05
FARSB	-0.453438377	3.06E-05
DGCR8	-0.453432643	3.06E-05
SLC12A6	0.453403818	3.06E-05
ACBD4	-0.453384928	3.07E-05
KLHL2	0.453382061	3.07E-05
ORM2	0.453363011	3.07E-05
DOK3	0.453333165	3.07E-05
TBKBP1	0.453331479	3.07E-05
DNAJC11	-0.453053276	3.11E-05
DNAJA3	-0.453018203	3.12E-05
NT5DC4	0.453018203	3.12E-05
EARS2	-0.452977402	3.12E-05
CNPY4	-0.45292682	3.13E-05
GALK1	0.452790582	3.15E-05
RBM25	-0.452783662	3.15E-05
CC2D2B	0.452775073	3.15E-05
BLTP2	-0.452714708	3.16E-05
CBX5	-0.4526992	3.16E-05
TMC6	-0.452446288	3.20E-05
STK3	0.452395706	3.21E-05
GET1	0.45238592	3.21E-05
CEBPB	0.452363489	3.21E-05
IARS1	-0.452345123	3.21E-05
N4BP2L2	-0.452335338	3.22E-05
DHX15	-0.452189308	3.24E-05
SH3PXD2A	-0.452168085	3.24E-05
CD47	-0.452057133	3.26E-05
GAREM2	-0.452015557	3.26E-05
DROSHA	-0.45200655	3.26E-05
SDAD1	-0.45200655	3.26E-05
ACSL6	-0.451940465	3.27E-05
CLIC5	-0.451829511	3.29E-05
MRPL52	-0.451766283	3.30E-05
CUL4A	-0.451753637	3.30E-05
KHDRBS1	-0.451746704	3.30E-05

FLYWCH1	-0.451712844	3.31E-05
LAMTOR5	0.451662262	3.31E-05
PIK3CD-AS1	0.451636971	3.32E-05
C1orf174	-0.451510515	3.34E-05
NSUN6	-0.451475433	3.34E-05
PLIN3	0.451399559	3.35E-05
LINC02363	0.451364476	3.36E-05
XRCC6	-0.451358768	3.36E-05
FAM177A1	0.451260456	3.38E-05
LOC101927178	0.451260456	3.38E-05
GCN1	-0.451257603	3.38E-05
USP14	-0.451207021	3.38E-05
PFDN1P2	0.451033751	3.41E-05
MRPS27	-0.45095696	3.42E-05
LINC02596	0.450928818	3.43E-05
NFAM1	0.450927577	3.43E-05
METTL3	-0.450881087	3.43E-05
RASSF2	0.450786948	3.45E-05
CNOT11	-0.45066896	3.47E-05
SCLT1	0.450564945	3.48E-05
DIDO1	-0.450542502	3.49E-05
TMEM42	-0.450451134	3.50E-05
ANAPC16	-0.450299386	3.52E-05
PNN	-0.450239005	3.53E-05
POMT1	-0.450198221	3.54E-05
NR2C1	-0.450170083	3.55E-05
TDP2	0.45016313	3.55E-05
ERMARD	-0.45009421	3.56E-05
TPRKBP2	0.450092948	3.56E-05
COX6B2	0.44994628	3.58E-05
ST3GAL5	-0.449866589	3.59E-05
DCAF7	-0.449856788	3.60E-05
CWC25	0.44979229	3.61E-05
FLOT2	0.449766998	3.61E-05
UBAP1	0.449689551	3.62E-05
CXCL6	0.449679749	3.62E-05
DDX18	-0.449613677	3.63E-05
STYXL1	0.449563095	3.64E-05
FRMD8	0.449515355	3.65E-05
PCID2	-0.449411348	3.67E-05
GATA3	-0.449386057	3.67E-05
LOC254896	0.449366448	3.68E-05
IL1R2	0.449360766	3.68E-05
RNF13	0.44932567	3.68E-05

PLAUR	0.44923431	3.70E-05
DCAF5	-0.449230186	3.70E-05
ERCC3	-0.449133145	3.71E-05
RN7SL172P	0.449107854	3.72E-05
ATP5F1B	-0.449106567	3.72E-05
TMEM123	-0.448936493	3.75E-05
DTX3	-0.448930816	3.75E-05
FKBP3	-0.448718678	3.78E-05
CENATAC	-0.448541639	3.81E-05
RNF115	-0.448541639	3.81E-05
SLC24A3	0.448500866	3.82E-05
SSH3	0.448424993	3.83E-05
RABGEF1	0.4483646	3.84E-05
TBRG1	-0.448326663	3.85E-05
GK	0.448266269	3.86E-05
DSC2	0.448250789	3.86E-05
SLC25A42	-0.448247955	3.86E-05
PLK2	0.448172081	3.88E-05
GAS5	-0.448149623	3.88E-05
TMEM119	0.448125854	3.88E-05
SLED1	0.448121499	3.89E-05
KMT2A	-0.448096208	3.89E-05
UFSP2	-0.448070916	3.89E-05
TMEM263	-0.448045625	3.90E-05
RBP7	0.447944461	3.92E-05
MIX23	-0.447843296	3.93E-05
EIF2B1	-0.447820836	3.94E-05
PAN2	-0.447820836	3.94E-05
MCCC2	-0.447770254	3.95E-05
RORC	-0.447681734	3.96E-05
SLC35A2	0.447640967	3.97E-05
PPIF	0.447590384	3.98E-05
RGL4	0.44748922	4.00E-05
SEMA3C	0.447454112	4.00E-05
EMC1	-0.447428821	4.01E-05
GALNS	0.447413346	4.01E-05
DTX2	0.447378238	4.02E-05
TMEM156	-0.447188553	4.05E-05
KCTD7	-0.447185726	4.05E-05
KCNJ15	0.447183055	4.05E-05
TMSB10	-0.447112679	4.06E-05
PPM1K	-0.44702416	4.08E-05
MEFV	0.44688654	4.11E-05
CAMK4	-0.446872412	4.11E-05

TMED8	0.446831649	4.12E-05
GOLGA7	0.446796538	4.12E-05
HSP90B1	-0.44676425	4.13E-05
CMPK1	-0.446755776	4.13E-05
SEPTIN1	-0.446730485	4.13E-05
PLK3	0.446654611	4.15E-05
PPP1R3E	-0.446559094	4.17E-05
CSDE1	-0.446512805	4.17E-05
LEF1	-0.446426991	4.19E-05
MMS19	-0.446404522	4.19E-05
PROK2	0.446394699	4.20E-05
VNN2	0.446278386	4.22E-05
P2RY13	0.446185464	4.23E-05
TUBB	-0.446116493	4.25E-05
BABAM2	0.445491218	4.37E-05
ATP6V1H	0.445364762	4.39E-05
MTRES1	-0.445256582	4.41E-05
RARA	0.445187724	4.43E-05
APEX1	-0.44511185	4.44E-05
NFE2L2	0.445021941	4.46E-05
ZNF133	-0.444988208	4.47E-05
SNHG32	-0.444899688	4.48E-05
C22orf46P	-0.444858939	4.49E-05
TAF1C	-0.444851918	4.49E-05
CLC	-0.444823814	4.50E-05
GLA	0.444785877	4.51E-05
INSR	0.444785877	4.51E-05
LMBRD1	0.444760586	4.51E-05
ARF5	0.4446819	4.53E-05
EXOSC9	-0.4446819	4.53E-05
TAF15	-0.444621484	4.54E-05
SYN2	0.444516105	4.56E-05
RPL14	-0.44437419	4.59E-05
NAIF1	0.44436857	4.59E-05
PCNT	-0.444343279	4.59E-05
CXCL5	0.444343279	4.59E-05
SLC6A6	0.444341867	4.59E-05
TMEM91	0.444333443	4.60E-05
EIF3F	-0.444302533	4.60E-05
SPOCK2	-0.444267405	4.61E-05
MTERF3	-0.44425476	4.61E-05
RNF130	0.444118448	4.64E-05
RPS6	-0.444090366	4.65E-05
NOL11	-0.444074912	4.65E-05

RPL5	-0.444017299	4.66E-05
BRD2	-0.444013076	4.66E-05
EIF5A	-0.443900681	4.68E-05
LOC102723566	-0.443900681	4.68E-05
USP24	-0.443827613	4.70E-05
DNAJB6	0.443771418	4.71E-05
KATNB1	0.443723642	4.72E-05
NEDD8	0.443647768	4.74E-05
CLUAP1	-0.443533957	4.76E-05
NLRC3	-0.44349602	4.77E-05
CENPBD2P	0.443442633	4.78E-05
RPL23AP79	0.443442633	4.78E-05
PISD	0.443392051	4.79E-05
WDR86	-0.443276554	4.82E-05
IMPDH1	0.443268398	4.82E-05
MXD3	0.443258555	4.82E-05
PRRC2C	-0.44325008	4.82E-05
EXOSC10	-0.443215013	4.83E-05
PABPN1	-0.443189722	4.83E-05
RNF34	0.443113848	4.85E-05
IFNGR2	0.443106806	4.85E-05
TOMM20	-0.443012684	4.87E-05
PEX26	-0.443012684	4.87E-05
SNAPC3	-0.442964902	4.88E-05
C5AR2	0.442929766	4.89E-05
KCNE3	0.4428286	4.91E-05
MYBBP1A	-0.442810354	4.91E-05
SDF2	0.442734481	4.93E-05
TG	0.442724634	4.93E-05
RPL17	-0.442714788	4.94E-05
APC	0.442610823	4.96E-05
MTDH	-0.442459075	4.99E-05
FAR2	0.442430987	5.00E-05
CPSF6	-0.442421138	5.00E-05
GALNT12	-0.442152784	5.06E-05
CD55	0.442151321	5.06E-05
KIF1B	0.442104997	5.07E-05
POLR2H	-0.442054414	5.08E-05
STRN4	0.442051619	5.08E-05
NELL2	-0.442016477	5.09E-05
CRADD	0.442001037	5.09E-05
NUP205	-0.441975746	5.10E-05
PRPF38A	-0.441798708	5.14E-05
ACVR1B	0.441773416	5.14E-05

LAS1L	-0.441748125	5.15E-05
SEPTIN11	-0.441725627	5.15E-05
IFRD1	0.441722834	5.15E-05
H3-3B	0.441593654	5.18E-05
GNAQ	0.441538734	5.20E-05
CNKSR2	-0.441306836	5.25E-05
MARCHF9	-0.441232446	5.27E-05
PEBP1	-0.441156572	5.28E-05
ITGB7	-0.441156572	5.28E-05
IKZF1	-0.441112868	5.29E-05
PPIE	-0.441004824	5.32E-05
RPGR	0.440938808	5.33E-05
MIR4435-2HG	0.440913517	5.34E-05
ZNF281	0.440865722	5.35E-05
BCL9L	-0.440862935	5.35E-05
PABIR2	-0.440812352	5.36E-05
CNDP2	-0.440751911	5.38E-05
RPL11	-0.4406381	5.40E-05
METRNL	0.440610023	5.41E-05
RPL7A	-0.440586015	5.42E-05
MSRA	0.440432985	5.45E-05
RNF213	-0.440424402	5.46E-05
LRIF1	0.44033182	5.48E-05
FNIP1	0.44033182	5.48E-05
RNF181	0.440306529	5.48E-05
NCOA1	0.44024179	5.50E-05
KLF7	0.440220793	5.50E-05
USB1	0.440205364	5.51E-05
PUS1	-0.440205364	5.51E-05
GLB1	0.440132274	5.52E-05
HADH	-0.440078908	5.54E-05
BMI1	-0.440053617	5.54E-05
ABCB8	-0.440028326	5.55E-05
CEP70	-0.440008599	5.55E-05
PFKFB3	0.439980526	5.56E-05
KCNN4	-0.439955234	5.57E-05
EXOSC2	-0.439952453	5.57E-05
BMT2	0.439929943	5.57E-05
ANXA6	-0.439904652	5.58E-05
RPS20	-0.439828778	5.60E-05
RPAP2	-0.439800706	5.61E-05
PELO	0.439750123	5.62E-05
ST3GAL2	0.439727612	5.62E-05
STK16	0.439547794	5.67E-05

NDUFB3	0.439497212	5.68E-05
CBLN3	-0.439388958	5.71E-05
GDPD3	0.439335597	5.72E-05
WWC3	0.439300438	5.73E-05
SLC39A13-AS1	0.439269591	5.74E-05
ABCC2	0.439183849	5.76E-05
HCAR3	0.439168427	5.76E-05
ZNF350	0.439117844	5.77E-05
C10orf143	-0.439110751	5.78E-05
NAA25	-0.439072814	5.79E-05
LINC01163	-0.439071007	5.79E-05
NSUN5P1	-0.439067262	5.79E-05
TM6SF1	0.43901668	5.80E-05
NAA80	-0.43901668	5.80E-05
CD74	-0.438972881	5.81E-05
MTERF4	-0.438940806	5.82E-05
ANXA3	0.438791833	5.86E-05
LOC100289473	0.438763768	5.86E-05
HEATR5A	0.438741251	5.87E-05
ARHGEF1	-0.438662603	5.89E-05
IL10RB	0.438642859	5.89E-05
AIG1	0.438637312	5.90E-05
NOXRED1	0.43853892	5.92E-05
DENND5A	0.438516402	5.93E-05
PTTG2	0.438471363	5.94E-05
LYSMD1	0.438440527	5.95E-05
WDFY3	0.438359109	5.97E-05
CFLAR	0.438321348	5.98E-05
YWHAQ	-0.4382058	6.01E-05
COX20	-0.43815678	6.02E-05
DHTKD1	0.437947343	6.07E-05
COQ6	-0.437903868	6.09E-05
TP53I11	0.437903868	6.09E-05
HLA-DPA1	-0.437903868	6.09E-05
TRBV5-5	-0.437855786	6.10E-05
MED30	0.437827995	6.11E-05
SNRNP200	-0.437805472	6.11E-05
CD247	-0.437782949	6.12E-05
GPR89B	-0.437742243	6.13E-05
ALPK1	0.437707074	6.14E-05
ANAPC4	-0.437633967	6.16E-05
GPR108	0.437625665	6.16E-05
NUP107	-0.437482217	6.20E-05
GYG1	0.437448627	6.21E-05

EEF2	-0.437404759	6.22E-05
ANO9	-0.437372754	6.23E-05
LYRM7	-0.43729688	6.25E-05
ZFTA	-0.43729688	6.25E-05
PHC1	-0.437221007	6.27E-05
FASTK	-0.437195716	6.27E-05
FRAT2	0.43718701	6.28E-05
TRIB2	-0.437145133	6.29E-05
RSAD1	-0.437084669	6.30E-05
TBCC	0.437077551	6.31E-05
VPS45	-0.43706926	6.31E-05
SNORD14E	-0.437042693	6.32E-05
BUB3	-0.437018678	6.32E-05
GPER1	0.436978151	6.33E-05
CYB5B	-0.436786697	6.39E-05
CRNKL1	0.436765766	6.39E-05
CALR	-0.436689892	6.41E-05
CLEC2D	-0.43663931	6.43E-05
DCTN4	0.436563437	6.45E-05
UBASH3B	0.436515614	6.46E-05
MPZL1	0.436477677	6.47E-05
ARHGAP30	-0.436369621	6.50E-05
IKZF3	-0.436338575	6.51E-05
YPEL5	0.436127499	6.57E-05
RPL13	-0.436101065	6.58E-05
PTPRE	0.436068642	6.59E-05
OSTF1	0.436037836	6.59E-05
RPS25	-0.43585804	6.65E-05
FAM110B	0.435696401	6.69E-05
SCO1	-0.435668355	6.70E-05
TRBC2	-0.435643064	6.71E-05
TSC22D3	0.435555668	6.73E-05
CIITA	-0.435526499	6.74E-05
TMEM184B	0.435516607	6.74E-05
MPRIP	-0.435425334	6.77E-05
TOPORS	0.435400043	6.78E-05
CDC42EP4	0.435374752	6.79E-05
ZMAT5	0.435149883	6.85E-05
CYLD	-0.435102051	6.87E-05
UCKL1	-0.435048717	6.88E-05
PLXNA3	-0.435020676	6.89E-05
ZNF32	-0.435020676	6.89E-05
MAN2C1	-0.43501078	6.89E-05
WDR43	-0.434972843	6.90E-05

NCSTN	0.434912365	6.92E-05
MPP7	0.434868929	6.93E-05
CS	-0.434859032	6.94E-05
RBM3	-0.434798553	6.95E-05
DYRK2	-0.434793055	6.96E-05
NPIPP1	-0.434767764	6.96E-05
DOCK8-AS1	0.434694639	6.99E-05
TMEM92-AS1	0.434681993	6.99E-05
PDCD4	-0.434656702	7.00E-05
RALA	-0.434616017	7.01E-05
VPS8	0.434565435	7.02E-05
NLRC4	0.434489561	7.05E-05
CCT3	-0.434479663	7.05E-05
NPTN	0.434368598	7.08E-05
TSR1	-0.434315269	7.10E-05
PYCARD	0.434264686	7.12E-05
OGFOD1	-0.434261941	7.12E-05
GPSM2	0.434261941	7.12E-05
PATJ	-0.434160776	7.15E-05
LPIN1	-0.434075001	7.17E-05
PRSS33	-0.433935082	7.22E-05
TAF1D	-0.433925997	7.22E-05
SNRPA1	-0.43384738	7.24E-05
PHYKPL	-0.433812185	7.25E-05
LINC03052	-0.43366955	7.30E-05
TSPAN2	0.433654953	7.30E-05
WDR36	-0.433654953	7.30E-05
RAB5B	0.433645049	7.31E-05
SEC24D	0.433645049	7.31E-05
RNASEH2B	-0.433629661	7.31E-05
CFAP45	0.433629661	7.31E-05
SUGT1	-0.433607112	7.32E-05
MESD	-0.43360437	7.32E-05
KRR1	-0.433528497	7.34E-05
GALT	-0.433528497	7.34E-05
TIPARP	0.433503206	7.35E-05
NBPF14	0.433503206	7.35E-05
SNORA77	0.433451375	7.37E-05
ADARB1	-0.433427332	7.37E-05
SPIN3	-0.433022674	7.50E-05
SREK1	-0.433012766	7.51E-05
NDUFA9	-0.432987474	7.51E-05
UHMK1	-0.432987474	7.51E-05
GRAMD1A	0.432942367	7.53E-05

FBXO38	0.432924246	7.53E-05
OSGEP	-0.432921509	7.53E-05
VTI1A	0.432810435	7.57E-05
MRPL33	0.432795053	7.58E-05
TRAJ5	-0.432757332	7.59E-05
WDCP	-0.432744471	7.59E-05
MINAR1	-0.432607432	7.64E-05
GARIN1A	0.432542141	7.66E-05
RPS8	-0.432477206	7.68E-05
RPAIN	-0.432466268	7.68E-05
MDH1	-0.432443711	7.69E-05
WIPI1	0.43228923	7.74E-05
GBP4	-0.43228923	7.74E-05
PRKCQ-AS1	-0.43228923	7.74E-05
CYBB	-0.432128904	7.79E-05
ICAM1	0.43203905	7.82E-05
EIF5	-0.432002125	7.83E-05
LOC105377267	-0.431989785	7.84E-05
PRPF3	-0.43195053	7.85E-05
GPR107	0.43192797	7.86E-05
TRBJ1-6	-0.431901155	7.87E-05
GIMAP5	-0.431738284	7.92E-05
CAMKK2	0.431684971	7.94E-05
MYO19	-0.431684971	7.94E-05
TANC2	0.431609097	7.97E-05
ING5	-0.431581077	7.98E-05
C10orf105	0.431581077	7.98E-05
URB1	-0.431530495	7.99E-05
RAD51D	-0.431479912	8.01E-05
NR1D2	-0.431378748	8.04E-05
DDX46	-0.431374285	8.05E-05
FBXO33	0.431353457	8.05E-05
IMPA2	0.431318247	8.07E-05
PHF19	-0.431277583	8.08E-05
ADAT2	-0.431277583	8.08E-05
SLC35G5	-0.431166235	8.12E-05
RNF207	-0.431120611	8.13E-05
ELP2	-0.43099938	8.17E-05
PLEKHM1	0.430872925	8.22E-05
LTBP3	-0.430774484	8.25E-05
SDHB	0.43077176	8.25E-05
LINC02288	0.430734795	8.27E-05
JPT2	-0.430648027	8.30E-05
HUWE1	-0.430645304	8.30E-05

MCM7	-0.430645304	8.30E-05
ANKRD40	-0.430638104	8.30E-05
SLC12A2-DT	-0.430620013	8.31E-05
ZFP90	-0.430594722	8.32E-05
NOP14	-0.430569431	8.32E-05
NARF	0.430536938	8.34E-05
MTOR	-0.430499001	8.35E-05
TRIM32	-0.430493557	8.35E-05
CTBP1	-0.430312036	8.42E-05
AOAH-IT1	0.430309315	8.42E-05
HNRNPF	-0.430185578	8.46E-05
TENT4A	-0.430104263	8.49E-05
RNU6-646P	0.430069181	8.50E-05
OLIG2	-0.430038863	8.51E-05
HSPH1	-0.430038316	8.51E-05
MCEMP1	0.429987734	8.53E-05
MRNIP	-0.429785404	8.60E-05
SH2D4B	0.429731484	8.62E-05
RAB35	0.429712248	8.63E-05
SYMPK	-0.429651736	8.65E-05
NARS1	-0.42964902	8.65E-05
THOC1	-0.429636374	8.66E-05
ZNF239	-0.429557784	8.69E-05
FLNB	-0.429484626	8.71E-05
USP10	0.429297656	8.78E-05
GPR183	-0.42925429	8.80E-05
SMNDC1	0.429228999	8.81E-05
RPL23AP21	0.429206421	8.82E-05
LOC127239154	0.429206421	8.82E-05
ARL8A	0.429171198	8.83E-05
VMP1	0.429156816	8.84E-05
TWNK	-0.429127834	8.85E-05
DICER1-AS1	0.429127834	8.85E-05
UQCRC2	-0.429117902	8.85E-05
TMEM252	0.428940863	8.92E-05
HPCAL4	-0.428925505	8.92E-05
LONRF3	0.428915571	8.93E-05
B4GAT1-DT	-0.42891556	8.93E-05
LZTS2	-0.428900214	8.93E-05
MTX3	-0.428849631	8.95E-05
MRPL44	0.42880176	8.97E-05
PDCD5	-0.428799049	8.97E-05
KPNA5	-0.428789115	8.97E-05
PDP2	-0.428774668	8.98E-05

LCK	-0.428748467	8.99E-05
KAT2A	-0.428713241	9.00E-05
MYCBP	0.428675304	9.02E-05
HP1BP3	-0.428640077	9.03E-05
MBOAT1	0.428548847	9.06E-05
GOLGA3	-0.428475682	9.09E-05
EEIG1	-0.428470264	9.09E-05
SETDB2	-0.42842239	9.11E-05
GPATCH4	-0.42839439	9.12E-05
RRP15	-0.428343808	9.14E-05
SUMF2	-0.428267935	9.17E-05
PIK3R1	-0.428238121	9.18E-05
CDH26	0.428235413	9.18E-05
MAVS	-0.42822006	9.19E-05
PRDM15	-0.428192061	9.20E-05
RAB32	0.428083664	9.24E-05
FCHO2	0.428065605	9.25E-05
TRMU	-0.427995144	9.28E-05
OSER1-DT	0.427989732	9.28E-05
CRYZ	-0.427939149	9.30E-05
RPS18	-0.427726879	9.38E-05
FLT3	0.427660947	9.41E-05
FUT8	-0.427651005	9.41E-05
HLX	0.427559782	9.45E-05
B3GNT8	0.427559782	9.45E-05
VSIR	0.427498294	9.47E-05
ITGA4	-0.427486611	9.48E-05
ORMDL1	-0.427448674	9.49E-05
LMAN1	-0.427423383	9.50E-05
SNX10	0.427408035	9.51E-05
ZC3H14	-0.427332162	9.54E-05
TMEM71	0.427284281	9.56E-05
ZNF248	-0.427132533	9.62E-05
CHMP2A	0.42707925	9.64E-05
RPL37	-0.427031368	9.66E-05
NIT2	-0.427006076	9.67E-05
STAT5B	0.426922812	9.70E-05
LTBR	0.426902212	9.71E-05
ITK	-0.426902212	9.71E-05
UBA6-DT	-0.426793799	9.75E-05
SECTM1	0.42673057	9.78E-05
PHF12	0.426664643	9.81E-05
NCOR1	-0.426609507	9.83E-05
SIRPA	0.42658961	9.84E-05

MDFIC	-0.426371097	9.93E-05
RBBP7	-0.426361147	9.93E-05
STX16	-0.426214789	9.99E-05
SLC31A1	0.426067603	0.000100548
ZNF74	-0.426067603	0.000100548
TMEM120A	0.426067603	0.000100548
QRICH1	-0.426067603	0.000100548
DDX5	-0.426020315	0.000100746
ABHD15	-0.425966439	0.000100973
SLC29A1	-0.425905904	0.000101227
TUT1	-0.425839983	0.000101505
PAQR8	-0.425839983	0.000101505
AIMP1	-0.425754156	0.000101869
CKLF	0.425713527	0.000102041
MYCBP2	-0.425637653	0.000102364
HDAC4	0.425637653	0.000102364
NVL	-0.425612362	0.000102471
TCF12	-0.425587071	0.000102579
RASGRP1	-0.425587071	0.000102579
RAB20	0.425511198	0.000102903
MINCR	-0.425488597	0.000103
GLS	-0.42545066	0.000103163
TSHZ3	0.425410033	0.000103337
FMNL3	-0.42533416	0.000103663
ZNF175	-0.42513183	0.000104538
RAB5IF	0.425081248	0.000104758
GRB10	0.424980083	0.000105199
NOP53	-0.424954792	0.000105309
TP53	-0.424919542	0.000105463
ALDH18A1	-0.42478044	0.000106074
DOCK4	0.424616046	0.000106799
PPP2R2A	0.424568148	0.000107012
DENND11	-0.424565463	0.000107024
MDC1	-0.424489589	0.000107361
IL2RB	-0.424451652	0.00010753
PHAF1	0.424423678	0.000107654
NDUFAF1	0.424347804	0.000107993
APOBEC3D	-0.424293018	0.000108238
UBE2Q2	-0.424287259	0.000108264
IL10RA	-0.424186094	0.000108719
SFXN5	0.424150838	0.000108878
ABLIM3	0.424019019	0.000109473
DEGS1	0.423993728	0.000109588
KANSL1L	0.423971117	0.00010969

EIF3E	-0.423892563	0.000110047
ASB8	0.423847341	0.000110254
YWHAB	-0.423810836	0.00011042
OAZ2	0.423794831	0.000110493
SYNJ2BP	-0.423768787	0.000110612
LMNB1	0.423766107	0.000110625
GOT2	-0.423715525	0.000110856
GPAT3	0.42361436	0.000111321
DDX51	-0.423607072	0.000111354
LDOC1	-0.423574491	0.000111504
MRPL32	-0.423513196	0.000111787
WBP1L	0.423503228	0.000111833
CYP17A1-AS1	0.423503228	0.000111833
STPG4	0.423502477	0.000111836
TLR5	0.42338674	0.000112372
SSR1	-0.423364126	0.000112477
ILKAP	-0.423331542	0.000112629
ATR	-0.423310866	0.000112725
TIA1	-0.423285575	0.000112843
CCDC50	-0.423123858	0.000113599
ZNF783	-0.423085921	0.000113777
HNRNPA3P2	0.423073275	0.000113836
XPO5	-0.422984756	0.000114252
MNT	0.422982081	0.000114265
ACSL5	-0.422782425	0.00011521
CDK14	0.422706551	0.00011557
CASS4	0.42268126	0.000115691
CD3G	-0.422678587	0.000115704
CAMP	0.422678587	0.000115704
ARL15	0.422653296	0.000115824
MTHFS	0.422567449	0.000116235
RPS24	-0.422552131	0.000116308
OGFOD3	-0.422542157	0.000116356
WDR6	-0.422456309	0.000116768
ATXN10	-0.422425676	0.000116915
EMG1	-0.422405726	0.000117011
TRAJ9	-0.422380308	0.000117134
CDKN2B	0.422375093	0.000117159
BRAP	0.422327181	0.00011739
TLE4	0.422273929	0.000117648
NSD2	-0.422172764	0.000118139
GLIS2	0.422134259	0.000118326
GALNT14	0.422071599	0.000118631
DNAJB12	0.422046308	0.000118755

RUSF1	-0.421985748	0.000119051
NUP188	-0.421970435	0.000119126
FSTL4	0.421942823	0.000119261
CELF1	-0.421884583	0.000119547
HLTF	-0.421641649	0.000120746
NPEPPS	0.42161171	0.000120895
ARIH2	-0.42161171	0.000120895
MCTP2	0.421606378	0.000120921
HNRNPAB	-0.421568441	0.00012111
PHAX	-0.421565776	0.000121123
TSPYL1	-0.421565776	0.000121123
SCART1	-0.421500563	0.000121448
TRBV2	-0.421489903	0.000121501
PYGL	0.421483946	0.000121531
GNLY	-0.421464611	0.000121627
SH2D1A	-0.421454631	0.000121677
EIF3B	-0.42143932	0.000121754
INO80	-0.421416694	0.000121867
BRIX1	-0.421404048	0.00012193
MTG1	-0.421363447	0.000122133
SLC38A1	-0.421338156	0.00012226
RTF2	0.421267609	0.000122615
CLINT1	-0.421264946	0.000122628
GTF2B	0.421262282	0.000122641
IL17RA	0.421235671	0.000122775
H2BC6	0.421186409	0.000123024
SGMS1	0.421176426	0.000123074
CCNG1	-0.421110535	0.000123407
H3-5	0.421110535	0.000123407
USP47	-0.421062615	0.00012365
ZNF512B	-0.421034662	0.000123792
CFAP70	0.420908206	0.000124435
EHMT2	-0.42078175	0.000125081
ANKMY1	-0.420731168	0.000125341
PJVK	-0.420687214	0.000125567
MKNK1	0.420663273	0.00012569
PTBP1	-0.420630003	0.000125861
CD3E	-0.42061269	0.00012595
FTH1	0.420571842	0.000126161
SETD6	-0.420528838	0.000126383
ZNF510	-0.420402382	0.000127039
PAXIP1-DT	-0.420402382	0.000127039
SERTAD1	0.4203518	0.000127302
NELFCD	-0.420329167	0.00012742

ZMYND11	-0.420265938	0.00012775
YIPF3	0.420253293	0.000127816
WWP1	-0.420149471	0.00012836
NLGN3	0.420126836	0.000128479
DAP3	-0.420048306	0.000128892
STAT4	-0.420038316	0.000128945
LINC00996	-0.419994826	0.000129174
MAN1A1	0.419873923	0.000129814
GADD45A	0.419845977	0.000129962
SNORD12C	0.419717301	0.000130647
RPS16	-0.419643647	0.00013104
APOL6	-0.419517192	0.000131718
F5	0.419441318	0.000132126
MIR646HG	0.419441318	0.000132126
LOC729296	0.419441318	0.000132126
PDPK1	0.419314862	0.00013281
RPL32	-0.419279577	0.000133001
MCPH1	-0.419102537	0.000133964
KDM2B	-0.41883433	0.000135436
SPOPL	0.418814335	0.000135546
ANKRD50	0.418783748	0.000135715
ZNF787	0.418685231	0.00013626
MIR22HG	0.418632001	0.000136556
GAS7	0.418612003	0.000136667
CYRIA	0.418574066	0.000136878
POR	0.418546128	0.000137033
ZNF697	0.418505545	0.00013726
PWWP3A	-0.418432317	0.000137669
LIAS	-0.418429672	0.000137684
KLF8	-0.418407026	0.000137811
PIH1D1	-0.418359089	0.00013808
MEGF9	0.418322311	0.000138286
ACSL3	0.418303216	0.000138394
MAP3K15	0.418165264	0.000139172
PTTG1IP	0.418116176	0.00013945
RNU6ATAC	0.418099433	0.000139544
TRIM39	0.418093528	0.000139578
EIF1B-AS1	0.418060878	0.000139763
METAP1D	-0.418050304	0.000139823
RPS14	-0.418027656	0.000139952
C6orf47	0.417974431	0.000140255
LINC01531	0.41793872	0.000140458
ST6GAL1	-0.417873266	0.000140832
GSTK1	-0.417873266	0.000140832

RDH16	-0.417791337	0.000141301
NDFIP1	-0.417772101	0.000141411
RFX5	-0.417749452	0.000141541
PARP6	-0.41774681	0.000141557
MAPK1	0.417669414	0.000142002
PDE8A	-0.417638281	0.000142181
STT3A	-0.41761299	0.000142327
MBOAT7	0.417595063	0.000142431
PDXDC1	-0.41751919	0.00014287
RYBP	0.417410658	0.0001435
MAP4K1	-0.417408019	0.000143515
ICE2	-0.417382727	0.000143662
ANKLE2	-0.417367443	0.000143751
PGAM5	-0.417342152	0.000143899
PNISR	-0.417196224	0.000144752
TBXAS1	0.417145097	0.000145052
PPP4R1-AS1	0.417077266	0.000145451
LETMD1	-0.417063949	0.000145529
RN7SL600P	0.417038658	0.000145679
PRMT2	-0.41701864	0.000145797
TTC3	-0.417013366	0.000145828
CCNDBP1	0.416993348	0.000145946
CKMT2-AS1	-0.416980703	0.000146021
AKAP1	-0.416940129	0.000146261
CLTCL1	0.416886911	0.000146576
SLAMF1	-0.416836328	0.000146876
HS1BP3	0.416775736	0.000147237
ACE	-0.416748131	0.000147401
ING2	0.416737799	0.000147463
UBQLN1	-0.416735164	0.000147479
TRBV7-9	-0.416613976	0.000148203
RARG	-0.416535468	0.000148674
NSA2	-0.416484885	0.000148979
CIRBP	-0.416459594	0.000149131
TET3	0.416434303	0.000149284
SPTBN1	-0.41643167	0.000149299
SPOUT1	-0.41643167	0.000149299
DDX55	-0.416305214	0.000150064
SH2D3A	-0.416305214	0.000150064
EPM2AIP1	-0.416254631	0.000150371
TUBGCP5	-0.41622934	0.000150525
RPL4P1	-0.416174865	0.000150856
DCTD	-0.416136069	0.000151093
CPEB2	0.416128176	0.000151141

MYO15B	-0.41600172	0.000151914
ESF1	-0.415979059	0.000152053
EFCAB2	0.415976429	0.000152069
TOR1AIP2	0.415849973	0.000152847
OBSCN-AS1	-0.415831411	0.000152962
ZNF701	0.415824682	0.000153003
FAM209A	0.415774099	0.000153316
TRBC1	-0.415766711	0.000153361
SLC35A5	0.415766711	0.000153361
DNAJB6P1	0.415672935	0.000153942
CNKSR1	-0.415650982	0.000154079
NOD1	-0.415460587	0.000155265
NUDT16	0.415352028	0.000155946
NFATC3	-0.41534415	0.000155995
MOSPD2	0.41534415	0.000155995
NFATC2	-0.415293567	0.000156313
DCAF13	-0.415157091	0.000157175
GCSAM	-0.415091238	0.000157592
CASP4	0.415069525	0.00015773
CD4	-0.415030634	0.000157977
ARNT2	0.414834641	0.000159227
TMCO3	0.414787744	0.000159527
LIPN	0.414762453	0.00015969
NIPAL3	-0.414737162	0.000159852
MMADHC	0.414502137	0.000161369
CSF2RA	0.414454175	0.00016168
SEC61A1	-0.414408376	0.000161978
OSBPL11	0.414383085	0.000162142
JUNB	0.414309831	0.00016262
RNF103	0.414281921	0.000162802
BCL10	0.414274514	0.00016285
TBC1D9B	-0.414180756	0.000163464
ALOX5AP	0.414130174	0.000163796
VPS52	-0.414029009	0.000164462
NEU1	0.413953135	0.000164964
MMP25	0.41393352	0.000165093
MAB21L3	0.413897759	0.00016533
MED31	0.413851971	0.000165634
BICD2	0.413804005	0.000165953
ELP1	-0.413801388	0.00016597
ZMIZ2	-0.413776097	0.000166138
CCDC174	0.41370284	0.000166627
AGTPBP1	0.413670134	0.000166845
SOCS3	0.413652257	0.000166965

RN7SKP16	0.41362435	0.000167152
RPS27A	-0.413500509	0.000167983
PARP12	-0.413497895	0.000168
ADGRE3	0.413399344	0.000168664
EPS15L1	0.413310824	0.000169263
NUP88	-0.413219692	0.000169881
FAM78A	-0.413194401	0.000170053
FOXK1	-0.413194401	0.000170053
GLG1	-0.413022585	0.000171226
PXYLP1	0.413019974	0.000171244
C2CD2	-0.41296678	0.000171608
PITX1-AS1	0.412934884	0.000171827
ACTL6A	-0.412916198	0.000171956
CD2	-0.412890907	0.00017213
PRPF8	-0.412888704	0.000172145
SEC24C	-0.412883482	0.000172181
GINM1	0.412840324	0.000172478
EXOSC7	-0.412691187	0.000173509
FCRL3	-0.412539439	0.000174563
BMP6	0.412486248	0.000174934
CBLB	-0.412435666	0.000175288
MIAT	-0.412435666	0.000175288
SYNJ1	0.412410374	0.000175465
RHOG	0.412402944	0.000175517
NAXD	-0.412387691	0.000175623
LIG1	-0.412385083	0.000175642
TMUB2	0.412258627	0.00017653
LINC01814	0.412200612	0.000176939
TRAC	-0.412172714	0.000177136
IPMK	0.412157463	0.000177244
RNF122	0.41210688	0.000177602
ZNF84	-0.412033612	0.000178121
MAPK14	0.411894886	0.000179108
BORCS8	0.411881864	0.000179201
TXK	-0.411778095	0.000179944
ATP6V0E1	0.411760615	0.000180069
F12	0.411755408	0.000180106
LINC01730	-0.411743505	0.000180192
SELP	0.411702222	0.000180488
RUVBL1	-0.411666888	0.000180742
SLFN11	-0.411626348	0.000181034
ZMYND15	0.411601057	0.000181216
OGT	-0.411596219	0.000181251
SUPV3L1	-0.411578368	0.00018138

RORA	-0.411575766	0.000181399
ZBTB47	0.411550475	0.000181582
FKBPL	0.411525184	0.000181764
QRSL1	-0.41122169	0.000183972
CDC37L1-DT	-0.411174435	0.000184318
FCMR	-0.411171107	0.000184342
TLR2	0.411164015	0.000184394
ZMYM4	-0.41113837	0.000184582
THEM4	-0.411120525	0.000184713
ICOS	-0.411120525	0.000184713
ZNF813	-0.41104985	0.000185233
NOLC1	-0.411011912	0.000185512
INAFM2	0.411009314	0.000185531
RGS19	0.410994069	0.000185644
MYO9A	-0.410958731	0.000185905
ELP3	-0.41093344	0.000186091
SPRED2	0.410870211	0.000186559
NMT1	-0.410832274	0.000186841
UTRN	-0.410796935	0.000187103
LINC01238	-0.410784289	0.000187197
HMGCR	0.410715866	0.000187706
APBB1IP	0.410708753	0.000187759
VCPKMT	0.410680526	0.00018797
DDX50	-0.410667881	0.000188064
MPST	0.410665284	0.000188083
CPT2	0.410614702	0.000188461
THUMPD1	-0.410614702	0.000188461
CLNS1A	-0.410614702	0.000188461
ACSL1	0.410474747	0.00018951
FAM114A1	0.410414967	0.00018996
HDAC1	-0.410341688	0.000190513
KLF16	0.410339093	0.000190533
TRBV12-4	-0.410302082	0.000190812
NMT2	-0.410212637	0.00019149
DGKE	-0.410210043	0.00019151
NSUN2	-0.410187345	0.000191682
POLG2	-0.4101747	0.000191778
NDC1	-0.41013417	0.000192086
FKBP1C	0.410124117	0.000192163
NDUFB2	-0.410108878	0.000192279
BRI3	0.41008618	0.000192452
CDK10	-0.410035598	0.000192838
CD40LG	-0.410033005	0.000192858
SRPRB	-0.410033005	0.000192858

HCG27	0.410022952	0.000192935
ANXA9	0.410010306	0.000193031
CBR3	-0.409945431	0.000193528
NFX1	-0.409881258	0.00019402
CACNB1	-0.409830676	0.000194409
MIR3605	0.409821867	0.000194477
PDK1-AS1	0.409797921	0.000194661
GABPB2	-0.409780093	0.000194799
TASOR	-0.40970681	0.000195364
SNHG12	-0.409630936	0.000195952
LOC101929372	0.409609518	0.000196118
UPF3A	-0.409542417	0.000196639
ASB2	-0.409431195	0.000197506
GOLGA6L9	-0.409427212	0.000197537
RNPS1	-0.409426017	0.000197546
SBNO2	0.409403314	0.000197724
UBE2F	0.409365377	0.00019802
RBM12	-0.409322559	0.000198356
RBM8A	-0.409289503	0.000198615
GBGT1	0.40927427	0.000198735
RPS6KA1	0.409234038	0.000199051
MCM3	-0.409226275	0.000199112
MTSS1	-0.409223688	0.000199132
MSI2	-0.409175693	0.00019951
ZNF222	0.409147814	0.00019973
SFSWAP	-0.409122523	0.000199929
G2E3P1	0.409115051	0.000199988
RRM1	-0.409099819	0.000200109
MXD1	0.408884792	0.000201815
UMPS	-0.40884432	0.000202137
ZBTB25	-0.408768447	0.000202743
CRTC3	-0.408753494	0.000202863
POGK	-0.408750909	0.000202884
CD6	-0.408743156	0.000202946
CDC23	-0.408707803	0.000203229
LOC122526776	0.408694394	0.000203337
TMEM169	0.408641991	0.000203757
CPEB3	0.408556055	0.000204449
SOWAHD	0.408492827	0.000204959
NAIP	0.408464953	0.000205184
ZC3H12D	-0.408394244	0.000205757
WDR4	-0.408389079	0.000205799
DLEU7	0.408315787	0.000206394
DLEU1-AS1	0.408315787	0.000206394

DRG2	-0.408262623	0.000206827
CEP95	-0.408161459	0.000207652
AGO4	0.408153975	0.000207714
MGP	0.408116105	0.000208024
COQ4	-0.408116037	0.000208024
ACTR5	-0.408052809	0.000208543
ZNF76	-0.408050229	0.000208564
ADAM17	0.408024937	0.000208771
RIC8B	-0.407984421	0.000209105
TPM2	-0.407928931	0.000209562
TEP1	-0.407911126	0.000209709
ITPR3	-0.407807382	0.000210566
ATP6V1E1	0.407807382	0.000210566
CUX1	0.407797315	0.00021065
VRK3	0.407746733	0.000211069
KIAA1191	-0.407734087	0.000211174
ZNF121	-0.407731509	0.000211196
GIHCG	0.407721441	0.000211279
RRM2B	0.407703884	0.000211425
CEBPB-AS1	0.40768866	0.000211552
PNPLA1	0.407655636	0.000211827
NACA	-0.407526841	0.000212902
ZMYM3	-0.407521687	0.000212945
IER5L	0.407478597	0.000213306
IL21R	-0.407428015	0.00021373
LINC00402	-0.407276268	0.000215008
DMAC1	-0.407250977	0.000215221
CLN3	0.407175103	0.000215863
SNRNP40	-0.407073939	0.000216722
SMARCC2	-0.407048648	0.000216937
MPEG1	-0.407008359	0.000217281
GZMK	-0.406962702	0.00021767
DDX42	-0.406962702	0.00021767
DAPK2	0.40693741	0.000217886
AJAP1	-0.406927565	0.00021797
ADAMTS7P1	-0.406916852	0.000218062
CYP2J2	-0.406916468	0.000218065
TM9SF3	-0.406899473	0.000218211
NAMPT	0.406853873	0.000218601
SORT1	0.406745154	0.000219535
DDI2	-0.406618698	0.000220626
PLD1	0.406542824	0.000221283
CD5	-0.406542824	0.000221283
PTPRJ	0.406523295	0.000221452

MITD1	-0.406520103	0.00022148
GIMAP1	-0.406492242	0.000221722
KLLN	0.406466951	0.000221941
SLC17A9	-0.406459445	0.000222007
INO80E	-0.406444229	0.000222139
AFG3L1P	-0.406444229	0.000222139
PPP1R3F	-0.406431584	0.000222249
SMCO4	0.406416368	0.000222381
RAD23B	0.406305127	0.000223352
GLO1	-0.406292482	0.000223463
DERA	0.406264621	0.000223707
AHSA2P	-0.406254545	0.000223795
ENTR1	-0.406229253	0.000224017
SMARCA5	-0.406191316	0.000224349
B4GALT1	0.406181239	0.000224438
DAAM2	0.406163457	0.000224594
HCFC1	-0.406014277	0.000225909
DNAJC10	-0.405966261	0.000226333
ABCD2	-0.405935836	0.000226603
EPHX2	-0.405925757	0.000226692
LINC01278	-0.405910545	0.000226827
GFOD2	0.405811946	0.000227703
RAB33B	0.40580938	0.000227726
UBASH3A	-0.405758798	0.000228177
TYROBP	0.405715912	0.000228559
FGGY	0.405682925	0.000228854
C1R	0.405634907	0.000229284
HK2	0.405607051	0.000229533
CYTH2	0.40558176	0.00022976
LYRM1	0.405546387	0.000230078
FXN	-0.40550845	0.000230419
SUN2	-0.405485723	0.000230624
ATG14	-0.405480595	0.00023067
HIP1R	-0.405480595	0.00023067
CAMK1D	0.405404722	0.000231354
TRAF4	-0.405293474	0.000232361
TMEM127	0.405283391	0.000232452
CDC42BPG	-0.405280829	0.000232476
FAM111A-DT	-0.405258266	0.00023268
CUEDC1	0.405252975	0.000232728
CHST7	0.405192309	0.00023328
CHD1L	-0.405154372	0.000233625
VAV3	0.405126519	0.000233879
PWP1	-0.405075937	0.000234341

ARMC7	0.404964687	0.00023536
TNFRSF8	0.40492419	0.000235732
OTUD7B	-0.404901459	0.000235941
ZNF549	-0.404898899	0.000235964
DNAL1	-0.404850876	0.000236406
GNL2	-0.404825585	0.000236639
ADGRL1	-0.404812939	0.000236756
RAB9A	0.404797734	0.000236896
OSBPL6	0.404764916	0.000237199
CLDN10	0.404746998	0.000237365
ABCD4	-0.404570113	0.000239006
LRRFIP2	0.404522089	0.000239453
NMNAT2	0.404508168	0.000239583
METTL2A	-0.404418366	0.000240422
DCAF1	-0.404393075	0.000240659
ZNF429	0.404393075	0.000240659
SMYD2	-0.404342493	0.000241133
SLC9A7P1	0.404319758	0.000241346
ZNF287	-0.404317202	0.00024137
DHX37	-0.404317202	0.00024137
NBPF10	0.404317202	0.00024137
BRWD3	0.40425653	0.000241941
RPN2	-0.404241328	0.000242084
RSPH14	0.404175677	0.000242703
PCM1	-0.404142719	0.000243014
ZNF562	-0.404142719	0.000243014
SH3GLB1	0.404097246	0.000243444
ZNF30	-0.404092136	0.000243492
USPL1	-0.40406429	0.000243756
CDHR3	-0.404038999	0.000243996
PSME2	-0.403988417	0.000244476
KLC2	-0.403988417	0.000244476
PSIP1	-0.403978325	0.000244572
SNHG4	-0.403912543	0.000245198
TMEM139	-0.403875735	0.000245549
ELOVL4	-0.403839223	0.000245897
MADD	-0.40383667	0.000245922
IMPDH1P10	0.40381651	0.000246114
ZSWIM6	0.403811378	0.000246163
RAB40C	0.403786087	0.000246405
AGTRAP	0.403710214	0.000247132
NACC2	0.403533176	0.000248837
MTMR10	0.403523082	0.000248934
CLSTN1	-0.403507885	0.000249081

VASP	0.403399609	0.00025013
ZBTB49	0.403383979	0.000250281
TULP3	-0.403381429	0.000250306
RPL22	-0.403371334	0.000250404
SUSD3	-0.403371334	0.000250404
FBL	-0.403333397	0.000250773
SIT1	-0.403280264	0.00025129
LILRB3	0.403275372	0.000251338
LOC102725035	0.403275372	0.000251338
LOC107987425	0.403275372	0.000251338
LOC107987462	0.403275372	0.000251338
LSM10	0.403270168	0.000251389
PTDSS1	-0.403234781	0.000251734
DNHD1	-0.403209489	0.000251981
MRPL24	-0.403153808	0.000252525
SLC26A8	0.403128517	0.000252773
LSG1	-0.40311842	0.000252872
RPL23A	-0.403083032	0.000253219
LNPEP	-0.403067838	0.000253368
PSMB3	0.403055192	0.000253492
KLHL42	-0.402951479	0.000254513
ZNF26	-0.402900897	0.000255012
ARPC1B	0.402875605	0.000255262
OSGIN1	0.402870602	0.000255311
WDR89	-0.402865507	0.000255362
UBALD2	0.40274915	0.000256515
PTCD2	-0.402698567	0.000257018
MTHFD1	-0.402647985	0.000257521
IRAK3	0.402599948	0.000258001
EXOC6	0.402549366	0.000258506
RPS2	-0.40254682	0.000258531
TNFAIP8	-0.402513974	0.00025886
ALDH3A2	-0.402501328	0.000258987
GNPNAT1	-0.402445656	0.000259545
VSIG1	-0.402420364	0.000259799
CTC1	-0.402359681	0.00026041
SON	-0.402354667	0.00026046
CATIP-AS1	0.402344491	0.000260563
LOC122455342	0.402293909	0.000261073
BCL2A1	0.402268617	0.000261328
CD3D	-0.402200373	0.000262018
RBM23	0.40219783	0.000262044
KRT23	0.402144704	0.000262582
CDR2	-0.402142162	0.000262608

DNAJC19	-0.402142162	0.000262608
TMEM81	0.402119413	0.000262839
GIPR	-0.402040997	0.000263637
PCED1A	-0.401970207	0.000264358
ZNF66	0.401955019	0.000264514
KARS1	-0.401828563	0.000265808
CAMK2D	-0.401740043	0.000266718
KLHL12	0.40168946	0.000267239
H2BC5	0.401686921	0.000267265
LBH	-0.401686921	0.000267265
ARB2A	0.401676815	0.000267369
BCL2	-0.401636338	0.000267787
NUTM2A-AS1	0.401636338	0.000267787
CMTM7	0.401585756	0.00026831
MAK16	-0.401489668	0.000269306
RUFY3	-0.401436547	0.000269859
ARL14EP	-0.401434009	0.000269885
PIK3CG	0.401383427	0.000270412
VNN1	0.401307553	0.000271204
ZNF337	-0.40123168	0.000271998
ISY1	0.401206389	0.000272263
KRT8P12	0.401122942	0.00027314
TTC24	-0.401118054	0.000273191
RASGEF1A	0.401105224	0.000273326
DTNBP1	0.401054642	0.000273859
TMEM70	0.401054642	0.000273859
DNAJC5	0.401004059	0.000274393
VIPAS39	0.400935791	0.000275115
AMN1	0.400791618	0.000276646
PGAP6	0.400665162	0.000277995
CDC42SE2	-0.400541238	0.000279323
SPTSSB	-0.400500768	0.000279758
SLC2A11	-0.400488123	0.000279894
TYW3	-0.400450186	0.000280302
FNDC3B	0.400361666	0.000281257
MTF1	0.400336375	0.000281531
KPNA1	0.400328791	0.000281613
GCFC2	-0.400285792	0.000282078
RPL18A	-0.400285792	0.000282078
HAUS5	-0.400270615	0.000282243
MICAL3	-0.400169451	0.000283342
BTN3A3	-0.40014416	0.000283617
MAP4K5	-0.400121398	0.000283865
CYYR1	0.400118868	0.000283893

DIP2B	0.400098637	0.000284113
DERL1	-0.400070816	0.000284417
RN7SL473P	0.400068286	0.000284444

Supplementary Table 2. 55 differential neutrophil coexpressed genes in acute pancreatitis.

Gene	logFC	Average expression	t	*P*.Value	adj.*P*.Val	B
AHNAK	-2.2706	2.5745	-10.3573	3.03E-18	3.82E-15	22.6932
IKZF1	-0.5547	1.1342	-10.1006	1.23E-17	1.04E-14	21.2937
ETS1	-1.4623	1.5217	-9.0498	3.69E-15	9.62E-13	15.6023
IL7R	-1.5753	1.5862	-8.0049	9.66E-13	9.33E-11	10.0598
RGS2	1.1608	2.1843	7.5584	9.90E-12	6.97E-10	7.7492
CD3E	-0.5467	0.6380	-7.5029	1.32E-11	8.90E-10	7.4655
FLNA	-0.9067	1.6992	-7.2765	4.21E-11	2.45E-09	6.3144
HSP90AB1	-0.5580	0.8986	-7.2748	4.25E-11	2.47E-09	6.3055
HNRNPU	-0.7356	2.0397	-7.2220	5.56E-11	3.12E-09	6.0391
EEF2	-1.4467	2.2878	-7.1837	6.75E-11	3.67E-09	5.8463
TCF7	-0.5220	0.5440	-7.1405	8.41E-11	4.44E-09	5.6293
TRAC	-0.5333	0.6297	-7.0235	1.52E-10	7.34E-09	5.0440
RTN3	0.5339	1.6241	6.8288	4.03E-10	1.65E-08	4.0788
PYGL	1.0929	1.7826	6.5272	1.79E-09	6.04E-08	2.6073
FPR1	0.8941	2.4463	6.4069	3.21E-09	1.01E-07	2.0291
SDCBP	1.0338	3.1454	6.3366	4.52E-09	1.35E-07	1.6936
PTMA	-0.5078	1.0020	-6.2492	6.88E-09	1.95E-07	1.2785
PABPC1	-1.9293	4.7511	-6.2047	8.52E-09	2.34E-07	1.0685
RNF149	0.7720	2.4592	6.2025	8.61E-09	2.36E-07	1.0585

SAMHD1	-0.5566	1.5318	-6.1608	1.05E-08	2.79E-07	0.8624
SRRM2	-0.6729	1.5871	-6.0903	1.47E-08	3.74E-07	0.5322
SLC11A1	0.5198	0.8258	6.0496	1.78E-08	4.41E-07	0.3429
CD74	-1.5132	2.6169	-5.9810	2.46E-08	5.80E-07	0.0249
MMP25	0.6957	1.2652	5.8690	4.16E-08	9.06E-07	-0.4902
S100A11	0.7571	1.8014	5.7921	5.94E-08	1.23E-06	-0.8407
AQP9	1.6036	3.9678	5.6353	1.22E-07	2.31E-06	-1.5476
H3-3B	0.5583	2.4535	5.6315	1.24E-07	2.34E-06	-1.5644
ALPL	0.8099	1.0730	5.4768	2.51E-07	4.31E-06	-2.2504
FCGR2A	0.8703	2.8549	5.4650	2.64E-07	4.51E-06	-2.3023
VNN2	1.8222	3.2833	5.4572	2.74E-07	4.65E-06	-2.3368
OGT	-0.6453	1.3885	-5.4500	2.83E-07	4.78E-06	-2.3681
ACSL1	3.6808	5.8548	5.2078	8.27E-07	1.23E-05	-3.4163
NAMPT	2.6406	5.9131	5.0990	1.33E-06	1.85E-05	-3.8773
MGAM	0.9696	1.5508	4.9932	2.09E-06	2.76E-05	-4.3196
HSPA8	-0.5879	1.3792	-4.8828	3.34E-06	4.16E-05	-4.7744
SLC2A3	0.9307	1.6873	4.8261	4.24E-06	5.13E-05	-5.0060
IRAK3	0.5693	0.7501	4.8235	4.28E-06	5.17E-05	-5.0165
RPL19	-0.6843	1.4318	-4.8169	4.40E-06	5.28E-05	-5.0431
MAPK14	0.5631	1.0141	4.7942	4.84E-06	5.73E-05	-5.1348
CD55	0.6671	1.1606	4.5759	1.19E-05	1.25E-04	-6.0038
FCGR3B	2.9254	9.5157	4.5424	1.36E-05	1.41E-04	-6.1348
LOC124905743	2.9254	9.5157	4.5424	1.36E-05	1.41E-04	-6.1348

HNRNPH1	-0.5643	1.2878	-4.3164	3.33E-05	3.10E-04	-6.9994
TLR2	0.5007	1.3035	4.3163	3.33E-05	3.10E-04	-6.9996
PROK2	0.7319	1.0912	4.2856	3.76E-05	3.43E-04	-7.1146
IFITM2	0.7578	2.4648	4.0732	8.46E-05	6.96E-04	-7.8941
RPL13A	-0.6005	1.4198	-3.8121	2.21E-04	1.61E-03	-8.8104
RNF213	-0.6279	1.6953	-3.7463	2.80E-04	1.98E-03	-9.0342
BASP1	0.7283	1.7856	3.7430	2.83E-04	2.00E-03	-9.0452
RPL3	-0.6436	1.5896	-3.5911	4.82E-04	3.17E-03	-9.5493
DDX5	-0.6889	2.8182	-3.5798	5.01E-04	3.28E-03	-9.5861
RPL4	-0.5320	1.5432	-3.2183	1.67E-03	9.33E-03	-10.7137
MXD1	0.6018	3.6189	3.2157	1.68E-03	9.40E-03	-10.7217
NIBAN1	0.6026	2.8697	3.1370	2.16E-03	1.17E-02	-10.9537
EEF1A1	-1.3182	4.7433	-2.7515	6.88E-03	3.23E-02	-12.0194
